# CTCF acetylation at lysine 20 is required for the early cardiac mesoderm differentiation of embryonic stem cells

**DOI:** 10.1186/s13619-022-00131-w

**Published:** 2022-09-19

**Authors:** Shixin Gong, Gongcheng Hu, Rong Guo, Jie Zhang, Yiqi Yang, Binrui Ji, Gang Li, Hongjie Yao

**Affiliations:** 1 Center for Health Research, Joint School of Life Sciences, CAS Key Laboratory of Regenerative Biology, Guangzhou Institutes of Biomedicine and Health, Chinese Academy of Sciences, Guangzhou Medical University, Guangzhou, China; 2 Department of Life Sciences, Changzhi University, Changzhi, China; 3 Guangdong Provincial Key Laboratory of Stem Cell and Regenerative Medicine, GIBH-CUHK Joint Research Laboratory on Stem Cell and Regenerative Medicine, Guangzhou Institutes of Biomedicine and Health, Chinese Academy of Sciences, Guangzhou, China; 4 Institute of Stem Cell and Regeneration, Chinese Academy of Sciences, Beijing, China; 5 University of Chinese Academy of Sciences, Beijing, China; 6 Cancer Centre, Faculty of Health Sciences, MoE Frontier Science Centre for Precision Oncology, University of Macau, Taipa, Macau SAR, China

**Keywords:** CTCF, CTCF acetylation, CBP, HDAC6, Early cardiac mesoderm differentiation

## Abstract

The CCCTC-binding factor (CTCF) protein and its modified forms regulate gene expression and genome organization. However, information on CTCF acetylation and its biological function is still lacking. Here, we show that CTCF can be acetylated at lysine 20 (CTCF-K20) by CREB-binding protein (CBP) and deacetylated by histone deacetylase 6 (HDAC6). CTCF-K20 is required for the CTCF interaction with CBP. A CTCF point mutation at lysine 20 had no effect on self-renewal but blocked the mesoderm differentiation of mouse embryonic stem cells (mESCs). The CTCF-K20 mutation reduced CTCF binding to the promoters and enhancers of genes associated with early cardiac mesoderm differentiation, resulting in diminished chromatin accessibility and decreased enhancer-promoter interactions, impairing gene expression. In summary, this study reveals the important roles of CTCF-K20 in regulating CTCF genomic functions and mESC differentiation into mesoderm.

**Supplementary Information:**

The online version contains supplementary material available at 10.1186/s13619-022-00131-w.

## Background

Interphase chromosomes in eukaryotic cells are partitioned into discrete megabase-sized topologically associated domains (TADs), and the boundaries of TADs are enriched for the binding of the architectural protein CCCTC-binding factor (CTCF) (Dixon et al. 2012, Nora et al. 2012, Ong and Corces 2014). CTCF, which is ubiquitously expressed and highly conserved in eukaryotes (Filippova et al. 1996, Klenova et al. 1993), is composed of an N-terminal domain, a C-terminal domain, and a DNA-binding domain with 11 zinc fingers (Ghirlando and Felsenfeld 2016). CTCF is implicated in diverse regulatory functions, including transcriptional activation/repression, gene insulation, imprinting, X chromosome inactivation, and long-range DNA–DNA contacts (Phillips and Corces 2009). A number of CTCF cobinding partners have been identified and might promote CTCF loop formation (Hu et al. 2020). CTCF regulates loop extrusion of the cohesin complex, and cohesin progressively forms DNA loops but stalls at convergent CTCF sites (Fudenberg et al. 2016). The N-terminus of CTCF contains a YDF motif that interacts with a pocket formed by the SA2-SCC1 subunits of cohesin and is required for cohesin-mediated extrusion (Li et al. 2020). We recently identified a short CTCF isoform lacking the N-terminus and 2.5 zinc fingers of CTCF which is encoded by an alternatively spliced transcript skipping exons 3 and 4 (Li et al. 2019). CTCF genomic sites bound by the CTCF short isoform might fail to stop cohesin-mediated loop extrusion in the genome. These observations suggest that the N-terminus of CTCF plays a critical role in genome organization.

In addition to the important roles of CTCF in regulating gene expression and chromatin organization, posttranslational modifications of the CTCF protein also mediate its biological functions. Phosphorylated CTCF is present during interphase and mitosis, suggesting that CTCF phosphorylation may play different roles in different stages of the cell cycle (Xiang and Corces 2021). CTCF is phosphorylated at either S612 in the C-terminal region by casein kinase II (CKII) or at T374 and S402 in zinc finger linkers by LATS kinases (Klenova et al. 2001, Luo et al. 2020). Coexpression of CTCF with CKII causes the function of CTCF to switch from a repressor to an activator (El-Kady and Klenova 2005). The phosphorylation of CTCF at zinc finger linkers disables CTCF DNA-binding activity at sites highly enriched at the boundaries of chromatin domains containing LATS signaling pathway target genes (Luo et al. 2020). Multiple threonine and serine residues in CTCF are phosphorylated during mitosis (Del Rosario et al. 2019, Sekiya et al. 2017). CTCF is frequently phosphorylated at serine 224 in pericentric regions by Polo-like kinase 1 during the G2/M transition and affects the expression of hundreds of genes without affecting mitosis or chromatin organization (Del Rosario et al. 2019). CTCF is also poly(ADP)ribosylated, and poly(ADP)ribosylation regulates CTCF-dependent chromatin insulation (Yu et al. 2004). In *Drosophila*, CTCF poly(ADP)ribosylation is required to facilitate interactions between distant sites (Ong et al. 2013). In addition, lysine 74 and lysine 698 of the CTCF protein are SUMOylated, and although the small ubiquitin-like protein SUMO does not affect the ability of CTCF to bind to DNA in vitro, SUMOylation contributes to the repressive function of CTCF (MacPherson et al. 2009). In this study, we discovered a previously unidentified modification of acetylation in CTCF.

We found that CTCF, a substrate of CREB-binding protein (CBP), is acetylated at lysine 20 (K20). CTCF is deacetylated by histone deacetylase 6 (HDAC6). We also showed that CTCF acetylation is essential to maintaining the early mesoderm differentiation of mouse embryonic stem cells (mESCs). Loss of CTCF-K20 acetylation led not only to the selective dissociation of CTCF from a small subset of genes associated with early mesoderm differentiation but also to a reduction in chromatin accessibility, enhancer-promoter (EP) interactions, and gene expression. In summary, we revealed that CTCF-K20, which could be acetylated by CBP and deacetylated by HDAC6, is essential for mesoderm differentiation of mESCs.

## Results

### CTCF is acetylated at lysine 20

**Fig. 1:**
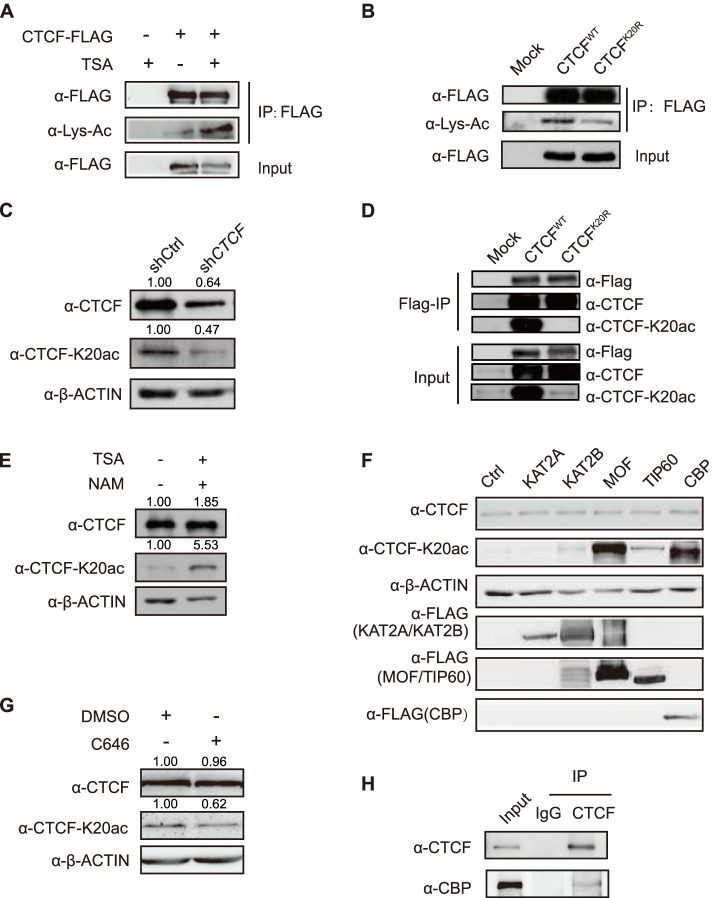
CTCF-K20 is acetylated by CBP. (**A**) Detection of the interaction between CTCF-Flag and the lysine-acetylated protein in HEK293T cells treated with TSA (10 μM) for 12 h. Membranes were immunoblotted with anti-Flag and anti-acetyl-lysine (Lys-Ac) antibodies after Flag co-IP experiments. (**B**) Detection of the interaction between Flag-tagged CTCF or the CTCF-K20R mutant and the lysine-acetylated protein in HEK293T cells. Membranes were immunoblotted with anti-Flag and anti-acetyl-lysine (Lys-Ac) antibodies after Flag co-IP experiments. (**C**) Western blots showing CTCF, CTCF-K20ac and β-ACTIN levels in control shRNA- and *CTCF* shRNA-depleted HEK293T cells. (**D**) Detection of the interaction between Flag-tagged CTCF or the CTCF-K20R mutant and CTCF-K20ac in HEK293T cells. Membranes were immunoblotted with anti-FLAG, anti-CTCF and anti-CTCF-K20ac antibodies after Flag co-IP experiments. (**E**) Western blots showing CTCF, CTCF-K20ac and β-ACTIN levels in HeLa-S3 cells treated with TSA (10 μM) and NAM (5 mM) for 12 h. (**F**) Western blots showing CTCF, CTCF-K20ac, Flag-tagged acetyltransferases and β-ACTIN levels in HeLa-S3 cells transfected with different constructs of Flag-tagged acetyltransferases. (**G**) Western blots showing CTCF, CTCF-K20ac and β-ACTIN levels in HEK293T cells treated with either the dimethyl sulfoxide (DMSO) control or the CBP inhibitor C646. (**H**) Detection of the interaction between CTCF and CBP using endogenous co-IP experiments. Membranes were immunoblotted with anti-CTCF and anti-CBP antibodies

To investigate whether CTCF could be acetylated in cells, we transfected a CTCF-Flag construct into HEK293T cells treated with trichostatin A (TSA) (Yoshida et al. 1990), a broad-spectrum HDAC inhibitor, and performed Flag-coimmunoprecipitation (co-IP) experiments. Our data indicated that CTCF-Flag pulled down more acetylated proteins from cells treated with TSA than that from cells without TSA treatment (Fig. 1A), suggesting that CTCF is acetylated in HEK293T cells. Mass spectrometry data indicated that lysine 20 of CTCF might be acetylated (from https://www.phosphosite.org/siteAction.action?id=3979892) (Hornbeck et al. 2015). The sequence analysis indicated that CTCF-K20 is evolutionarily conserved from zebrafish to mammals (Fig. S1A). Therefore, we generated a construct with a point mutation in CTCF in which lysine 20 was replaced with arginine 20 (K20R), and Flag-IP experiments indicated that the acetylation of the CTCF-K20R mutant was significantly reduced compared to that of CTCF-K20 (Fig. 1B). To assess the specificity and roles of CTCF-K20 acetylation, we generated an antibody that specifically recognized acetylated lysine 20 of CTCF (named the “anti-CTCF-K20ac antibody”). A dot blot assay indicated that the anti-CTCF-K20ac antibody specifically recognized the acetylated CTCF-K20 peptide but not the unmodified peptide (Fig. S1B). Furthermore, CTCF knockdown significantly reduced the CTCF-K20 acetylation level in HEK293T cells (Fig. 1C). A specific acetylated CTCF-K20 signal was detected in cells ectopically expressing wild-type (WT) CTCF but not the K20R mutant (Fig. 1D). Moreover, the combination of TSA with another broad-spectrum inhibitor, nicotinamide (NAM, an inhibitor of NAD + -dependent class III SIRTs) (Bitterman et al. 2002) increased the level of CTCF-K20 acetylation compared to the untreated cells (Fig. 1E). Together, these data indicated that CTCF can be acetylated at lysine 20 in cells.

### CTCF-K20 is mainly acetylated by CBP

To identify which acetyltransferases are critical for CTCF-K20 acetylation, we transfected individual histone acetyltransferase (KAT2A, KAT2B, MOF, TIP60, and CBP) into HEK293T cells, respectively, and then examined the effect of these histone acetyltransferases on CTCF-K20 acetylation. Our data indicated that both CBP and MOF significantly increased the CTCF-K20 acetylation level, and TIP60 and KAT2B increased CTCF-K20 acetylation slightly (Fig. 1F). However, KAT2A exerted no effects on CTCF-K20 acetylation (Fig. 1F). To confirm whether both CBP and MOF can specifically regulate CTCF-K20 acetylation, we treated cells with either the CBP inhibitor C646 (Bowers et al. 2010) or MOF short hairpin RNA (shRNA) vectors. Our results showed that CTCF-K20 acetylation was significantly inhibited by the CBP inhibitor C646 (Fig. 1G) but was not affected by MOF knockdown (Fig. S1C and S1D), suggesting that CBP, but not MOF, is a key acetyltransferase catalyzing CTCF-K20 acetylation. Co-IP experiments confirmed the endogenous interaction between CTCF and CBP (Fig. 1H). Taken together, these results showed that CBP might be a major enzyme catalyzing CTCF-K20 acetylation.

### CTCF-K20 is mainly deacetylated by HDAC6

**Fig. 2:**
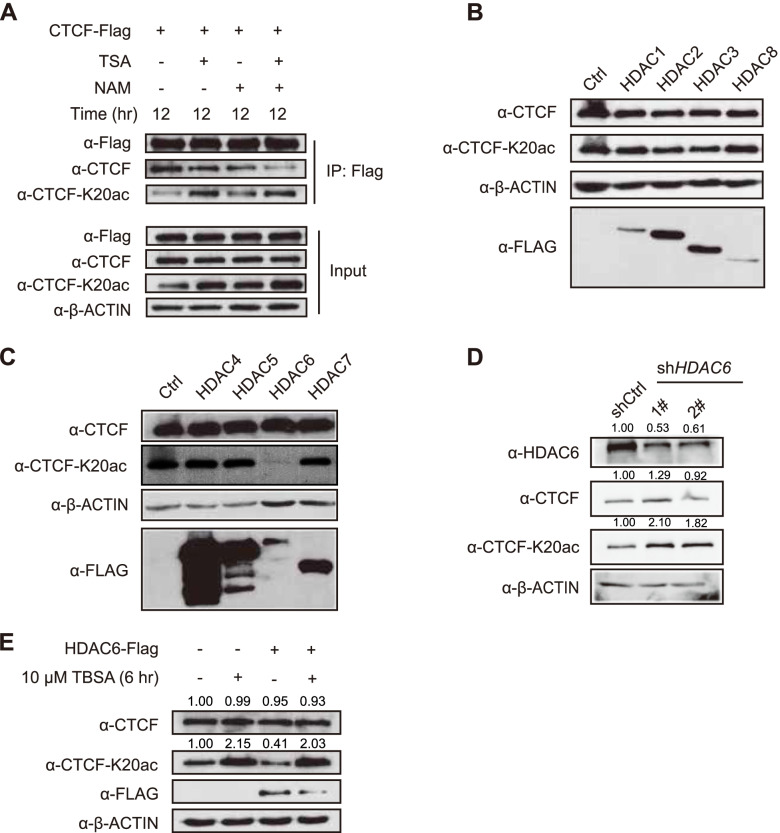
CTCF-K20 is deacetylated by HDAC6. (**A**) Detection of the levels of FLAG-CTCF and CTCF-K20ac in HEK293T cells treated with TSA (10 μM), NAM (5 mM) or both. Membranes were immunoblotted with the indicated antibodies after Flag co-IP experiments. (**B** and **C**) Western blots showing CTCF, CTCF-K20ac, β-ACTIN and FLAG-tagged HDAC levels in HEK293T cells transfected with different HDAC constructs, respectively. (**D**) Western blots showing HDAC6, CTCF, CTCF-K20ac and β-ACTIN levels in HEK293T cells transfected with the scrambled control shRNA or *HDAC6* shRNA. (**E**) Western blots showing CTCF, CTCF-K20ac, FLAG and β-ACTIN levels in HEK293T cells treated with 10 μM TBSA for 6 h

To identify the deacetylase critical for CTCF-K20 deacetylation, we treated HEK293T cells with TSA (an inhibitor of class I, II, and IV HDACs), NAM or both. Our data indicated that NAM slightly but TSA significantly increased CTCF acetylation (Fig. 2A), suggesting that HDACs might be the major deacetylases of CTCF-K20. We ectopically transfected 8 individual HDACs (*HDAC1* to *HDAC8*) into HEK293T cells to further explore which HDAC was involved in this process and found that overexpression of *HDAC6*, but not other HDACs, significantly decreased endogenous CTCF-K20 acetylation (Fig. 2B and 2C). *HDAC6* knockdown had little effect on CTCF expression but led to increased CTCF-K20 acetylation (Fig. 2D). Furthermore, treatment of HEK293T cells with tubastatin A (TBSA) (Butler et al. 2010), an HDAC6 inhibitor, reversed HDAC6 inhibition and increased CTCF-K20 acetylation (Fig. 2E). In summary, we concluded that HDAC6 is the deacetylase critical for CTCF-K20 deacetylation.

### CTCF-K20 is indispensable for proper cardiac differentiation of embryoid bodies

**Fig. 3:**
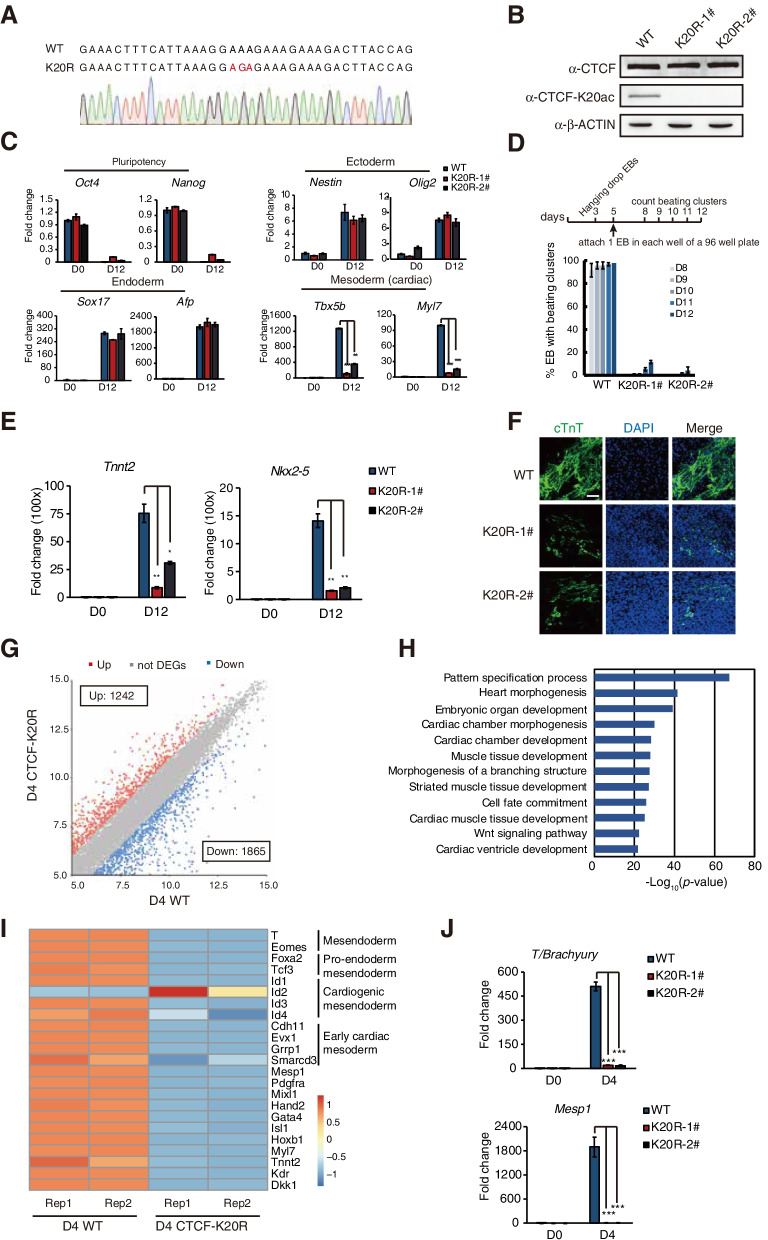
The CTCF-K20 mutation impaired the cardiac differentiation of embryoid bodies. (**A**) Chromatogram from Sanger sequencing showing the sequence of the mutated DNA. (**B**) Western blots showing CTCF, CTCF-K20ac and β-ACTIN levels in both WT and CTCF-K20R mESCs. (**C**) RT–qPCR analysis of the expression of pluripotency (*Oct4* and *Nanog*), ectoderm (*Nestin* and *Olig2*), endoderm (*Sox17* and *Afp*), and cardiac mesoderm (*Tbx5b* and *Myl7*) marker genes using RNA lysates from WT and CTCF-K20R mESCs and EBs at day 12 after mESC differentiation. Expression was normalized to *Gapdh*. (**D**) Semiquantitative analysis of beating EBs. The data are presented as the averages of three experiments. (**E**) RT–qPCR analysis of the expression of selected genes (*Tnnt2, Nkx2-5*) in WT and CTCF-K20R ESCs and EBs on day 12. (**F**) Immunostaining analysis of the cardiomyocyte marker cTnT after EB differentiation on day 12. The scale bar represents 100 μm. (**G**) Scatter plot showing DEGs between WT and CTCF-K20R EBs 4 days after mESC differentiation. Two independent RNA-seq experiments were performed for each sample. (**H**) GO analysis of the significantly downregulated genes shown in Fig. 3G. (**I**) Heatmap showing the expression of marker genes related to myocardial differentiation that were derived from a previous report (Cunningham et al. 2017). (**J**) RT–qPCR analysis of the selected genes in both WT and CTCF-K20R mESCs and EBs at day 4 after mESC differentiation. Bar graphs in (**C**), (**D**), (**E**) and (**J**) represent the mean ± s.d. (*n* = 3) and *P* values were determined by *t* test (**P < 0.01, ***P < 0.001)

CTCF is a highly conserved protein, and loss of maternal CTCF results in early embryonic lethality at the peri-implantation stage (Moore et al. 2012). Conditional deletion of *Ctcf* leads to different tissue defects during development (Gomez-Velazquez et al. 2017, Hirayama et al. 2012, Soshnikova et al. 2010). To evaluate the biological functions of CTCF-K20, we generated a CTCF point mutation (K20R) in mESC clones using clustered regularly interspaced short palindromic repeats (CRISPR)/Cas9 technology. The K20R mutation of CTCF was confirmed by DNA sequencing (Fig. 3A) and Western blotting (Fig. 3B). The CTCF-K20R mutation had no effect on the maintenance of mESC self-renewal (Fig. S2A and S2B). Embryoid bodies (EBs) simulate the development of embryos and are often used to detect pluripotency and differentiation potential (Evans and Kaufman 1981). We generated EBs from WT and CTCF-K20R ESCs to investigate whether CTCF-K20 is essential for ESC differentiation. The morphology of the EBs derived from CTCF-K20R ESCs was very similar to that of EBs derived from WT ESCs on day 12 (Fig. S2C). We observed that WT EBs were beating once per 2 ~ 3 s on day 8 after differentiation. However, EBs derived from the CTCF-K20R mutant showed the significant smaller size and loss of the beating phenotype (Supplementary Movie 1 and Movie 2), indicating that mutation of lysine 20 in CTCF suppresses the proper cardiac differentiation of mESCs. Furthermore, although CTCF-K20R had little effect on the expression of marker genes *Oct4* and *Nanog* (pluripotency), *Sox17* and *Afp* (endoderm), *Nestin* and *Olig2* (ectoderm) during the EB differentiation of mESCs, CTCF-K20R significantly reduced the expression of mesoderm markers (*Tbx5b* and *Myl7*) on day 12 after differentiation (Fig. 3C).

To better count the beating EBs, we took advantage of a new protocol to drive differentiation (Fig. 3D). Our data indicated that 90% of WT EBs were beating on day 8 after differentiation, while CTCF-K20R EBs were not beating. Furthermore, all WT EBs were beating on day 12 after differentiation, whereas few EBs derived from the CTCF-K20R mutant were beating (Fig. 3D). In addition, compared with the WT control, the cardiac-specific genes, *Tnnt2* and *Nkx2-5*, in CTCF-K20R cells were not properly expressed (Fig. 3E). Immunofluorescence staining indicated that cTnT expression was significantly reduced in cells expressing the CTCF-K20R mutation but not the WT control cells (Fig. 3F). In summary, we concluded that lysine 20 of CTCF is indispensable for the cardiac mesoderm differentiation of mESCs.

### CTCF-K20R mutation suppresses the differentiation of mESCs into early mesoderm cells

To investigate the possible mechanism by which CTCF-K20 regulates cardiac mesoderm differentiation of mESCs, we performed RNA sequencing (RNA-seq) experiments with both WT and CTCF-K20R mutant cells on day 4 after EB differentiation. We identified 1242 upregulated and 1865 downregulated genes in cells expressing the CTCF-K20R mutant compared to their counterparts expressed in WT cells (Fig. 3G). Gene ontology (GO) analysis showed that the downregulated genes were involved in the processes of heart morphogenesis and cardiac chamber morphogenesis (Fig. 3H). EB formation is a chaotic process of ESC differentiation; therefore, estimating whether the downregulated genes were specific to the mesoderm lineage is difficult. We compared our RNA-seq data obtained on day 4 with published RNA-seq data obtained from early cardiac-mesoderm precursor cells on day 3 (Alexanian et al. 2017, Ritter et al. 2019) to further examine the effect of the CTCF-K20R mutation on the mesoderm differentiation of mESCs. We noticed that 654 and 725 genes that were originally upregulated during cardiac differentiation were downregulated in CTCF-K20R mutant cells in our study, respectively; 389 and 405 originally downregulated genes involved in cardiac differentiation were abnormally upregulated in our data after CTCF-K20R mutation, respectively (Fig. S3A and S3B). Furthermore, GO analysis showed that abnormally downregulated genes were associated with mesoderm-related genes (Fig. S3C), suggesting that CTCF-K20 regulates the mesoderm differentiation of mESCs. Heatmap analysis of our RNA-seq data indicated that the CTCF-K20R mutation led to the significantly downregulated expression of the selected early mesoderm-related genes on day 4 after differentiation (Fig. 3I). The RT–qPCR analysis validated the attenuated expression of selected genes (*T/Brachyury*, *Mesp1*) in EBs differentiated from the CTCF-K20R mutant (Fig. 3J). As a reduction in *T/Brachyury* expression in CTCF-K20R mutant EBs is essential for initial mesoderm cell determination (Fig. 3J) (Herrmann et al. 1990), we concluded that CTCF-K20 is involved in the early mesoderm determination of mESCs.

### CTCF-K20 is necessary for proper CTCF occupancy at the genes associated with early cardiac mesoderm differentiation

**Fig. 4:**
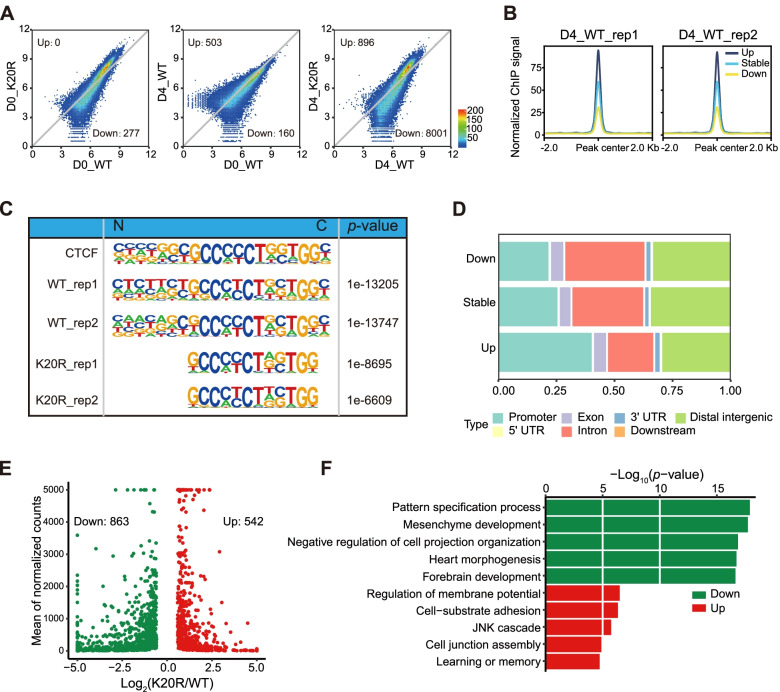
CTCF-K20R reduced CTCF binding in differentiated EBs. (**A**) Differential analysis of CTCF binding in both WT and CTCF-K20R mESCs and differentiated EBs at day 4 after differentiation. Two independent CTCF ChIP-seq experiments were performed for each sample. (**B**) The average binding strength for different types of CTCF sites using CTCF binding sites in WT EBs on day 4. (**C**) Motif analysis of CTCF binding sites in both WT and CTCF-K20R EB samples on day 4. The CTCF motif is derived from Homer. (**D**) Genomic distribution of CTCF binding sites in EB samples on day 4. (**E**) Scatter plot showing the DEGs located near the sites with decreased CTCF binding. (**F**) GO analysis of the DEGs shown in Fig. 4E. Green bars represent the top GO terms for the downregulated genes, and red bars represent the top GO terms for the upregulated genes

To further investigate the mechanism of CTCF-K20 in regulating early cardiac mesoderm differentiation of mESCs, we performed chromatin immunoprecipitation sequencing (ChIP-seq) experiments to identify CTCF binding sites in both mESCs and differentiated EBs on day 4 to assess whether CTCF-K20R affected the genome-wide distribution of CTCF in early cardiac mesoderm differentiation. We compared CTCF binding between mESCs and differentiated EBs and found that CTCF-K20R had little effect on CTCF binding on day 0 but significantly reduced CTCF binding in differentiated EBs compared to the corresponding WT cells on day 4 after differentiation (Fig. 4A). Compared to stable and upregulated CTCF sites, the reduced CTCF sites showed relatively weak binding strength (Fig. 4B). Motif analysis of CTCF peaks revealed that the topmost motif of CTCF binding sites in WT EBs on day 4 after differentiation was very similar to the conventional CTCF motif, while the topmost motif of CTCF binding sites in CTCF-K20R mutant EBs on day 4 after differentiation was much shorter (Fig. 4C). The missing motif for CTCF-K20R corresponds to the binding sequence of the N-terminal zinc fingers of CTCF, suggesting that CTCF-K20R mutation might be detrimental to the binding of CTCF.

The CTCF-K20R mutation led to retardation of early mesoderm determination of mESCs, which might be mainly caused by significantly reduced CTCF binding, as the reduced CTCF binding sites were predominant in the changed CTCF binding sites after CTCF-K20R mutation. Gene annotation of CTCF binding sites indicated that CTCF was mainly located in the promoter, intron, and intergenic regions (Fig. 4D). Genes located near the reduced CTCF binding sites were extracted and analyzed. Our results showed that a large number of genes were abnormally expressed (863 downregulated and 542 upregulated genes) on day 4 after differentiation (Fig. 4E). GO analysis showed that the downregulated genes, rather than the upregulated genes, were significantly involved in the process of heart development (Fig. 4F). These results suggested that decreased CTCF binding somehow regulates the expression of nearby genes and lead to the abnormal differentiation of mESCs.

### Reduction in CTCF binding and chromatin accessibility at the promoters is strongly correlated with decreased gene expression

**Fig. 5:**
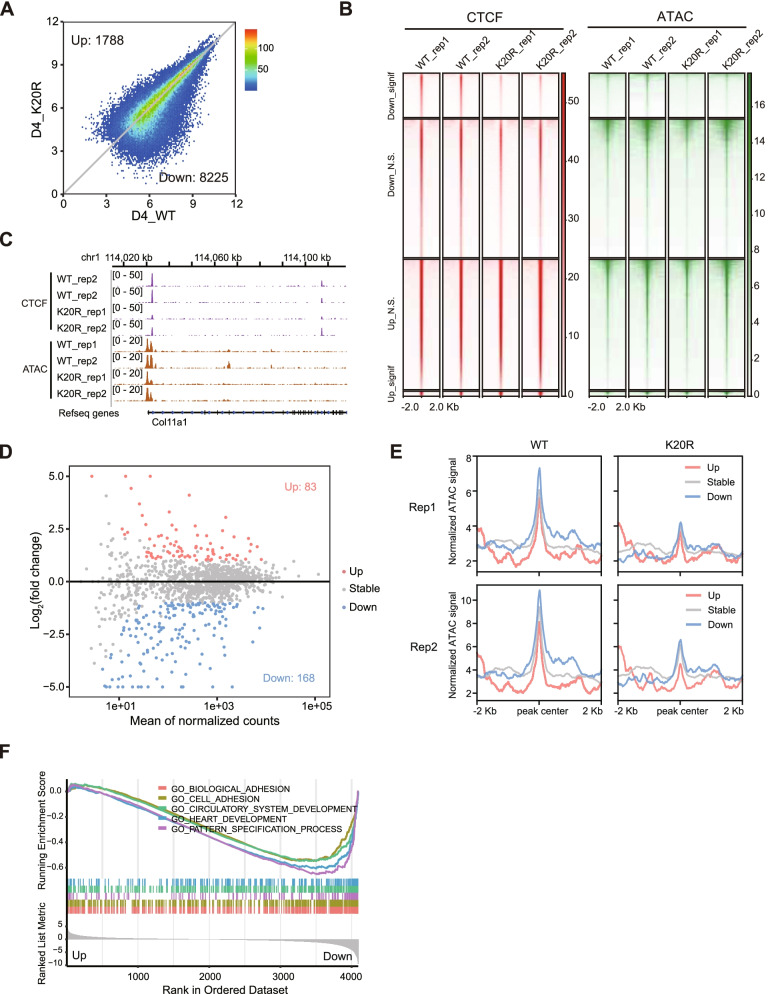
Relationship between reduced CTCF binding and chromatin accessibility. (**A**) Differential analysis of chromatin accessibility in both WT and CTCF-K20R differentiated EBs on day 4. Two independent ATAC-seq experiments were performed for each sample. (**B**) Heatmaps showing the CTCF binding signal and chromatin accessibility in both WT and CTCF-K20R EB samples on day 4 after differentiation. (**C**) Genome browser views of CTCF ChIP-seq and ATAC-seq data for selected loci in both WT and CTCF-K20R samples. (**D**) Scatter plot showing the expression change for genes with reduced CTCF binding in their promoters. (**E**) Comparison of chromatin accessibility at the promoters of genes shown in Fig. 5D in both WT and CTCF-K20R differentiated EBs on day 4. (**F**) GSEA analysis for the genes shown in Fig. 5D, which were ranked by log2 fold change comparing CTCF-K20R to WT differentiated EBs on day 4

As CTCF binding has been reported to modulate chromatin accessibility (Song et al. 2022), we performed an assay of transposase-accessible chromatin using high-throughput sequencing (ATAC-seq) experiments in both WT and CTCF-K20R cells, and found that chromatin accessibility was mainly reduced in CTCF-K20R cells on day 4 after differentiation compared with that in WT cells (Fig. 5A). An integrated analysis between CTCF ChIP-seq and ATAC-seq data showed that the significant decrease in CTCF binding to its target sites in CTCF-K20R mutant cells was consistent with the significant downregulation of the ATAC-seq signal (Fig. 5B and 5C), indicating that the downregulation of chromatin accessibility might be caused by the reduced CTCF binding. To test whether the reduced chromatin accessibility caused by the decreased CTCF binding impairs gene expression, we analyzed the expression of genes with decreased CTCF binding sites in their promoters and found that the downregulated genes were indeed more abundant than the upregulated genes (Fig. 5D), suggesting that decreased chromatin accessibility by CTCF binding reduction impaired gene expression. Furthermore, the ATAC-seq signal in the promoters of up-, stable- and downregulated genes, which exhibited decreased CTCF binding in their promoters, was analyzed, and results showed that the promoter regions of downregulated genes in CTCF-K20R mutant cells showed greater chromatin accessibility in WT EBs on day 4 of differentiation, and its downregulation might lead to a significant reduction of gene expression (Fig. 5E). Gene Set Enrichment Analysis (GSEA) indicated that these downregulated genes from panel 5D were significantly enriched in the process of heart development (Fig. 5F), indicating that CTCF bound to the promoters of many heart development-related genes, and CTCF-K20R mutation resulted in the reduction of CTCF binding, then led to reduced chromatin accessibility and further impaired the expression of heart development-related genes. Taken together, these data suggested that CTCF-K20 is required for proper CTCF binding to gene promoters of heart development-related genes, thereby facilitating the maintenance of chromatin accessibility of these genes.

### The change in gene expression induced by CTCF-K20R is not caused by a change in TAD boundaries with reduced CTCF sites

**Fig. 6:**
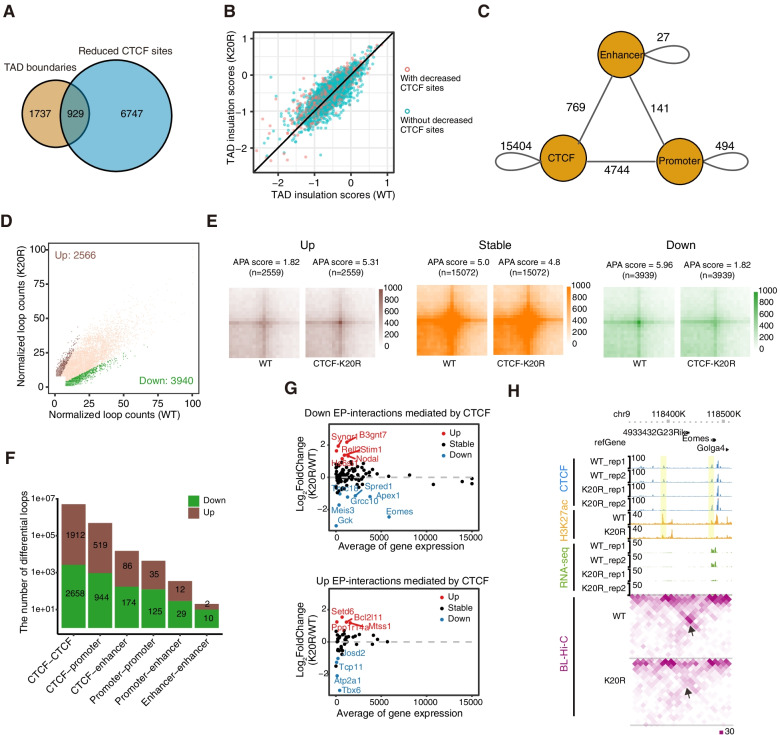
Differential CTCF binding in CTCF-K20R regulates EP interactions and their associated gene expression. (**A**) Venn diagram showing the overlap between the downregulated CTCF sites and TAD boundaries. Two independent BL-Hi-C experiments were performed for each sample. (**B**) Scatter plot showing the insulation scores of the TAD boundaries between the WT and CTCF-K20R mutant in EB samples on day 4 after mESC differentiation. Red points represent TAD boundaries with downregulated CTCF sites. (**C**) Categories of significant chromatin loops extracted from BL-Hi-C data. (**D**) Scatter plot showing the change in chromatin loops. Loops were considered changed if the loops were up- or down-regulated at least twofold. (**E**) Heatmaps showing the APA analysis for up-, stable- and down-regulated chromatin loops, respectively. (**F**) Categories of up- and downregulated chromatin loops. (**G**) Scatter plots showing the expression of genes associated with CTCF-mediated EP interactions. The gene expression level is represented as normalized read counts. (**H**) Representative genomic locus showing CTCF, H3K27ac, RNA-seq and BL-Hi-C data at the *Eomes* gene locus. Chromatin interactions are shown at 10 kb resolution. Yellow tracks represent enhancers and promoters. Black arrows point to the decreased EP interactions

CTCF plays an important role in regulating chromatin organization. To understand how downregulated CTCF binding affects the expression of cardiac differentiation-related genes, we performed bridge linker Hi-C experiments (BL-Hi-C) with WT and CTCF-K20R cells on day 4 after differentiation. The curves of contact frequency vs. distance decay showed no significant difference between WT and K20R mutant cells (Fig. S4A). The replicates of Hi-C datasets showed very high reproducibility (Fig. S4B) and were combined for subsequent analysis. The overall compartments exhibited little change in CTCF-K20R mutant cells compared with WT cells on day 4 after differentiation (Fig. S4C). TAD boundary analysis showed that a small number of sites with reduced CTCF binding were located in TAD boundaries (Fig. 6A). We compared the insulation scores at the TAD boundaries between WT cells and CTCF-K20R mutant cells on day 4 after differentiation and found that the insulation scores of most TAD boundaries with sites exhibiting decreased CTCF binding showed no significant change (Fig. 6B). We further chose two TAD boundaries with the altered insulation score for visual inspection and observed that these TAD boundaries contained several sites with decreased CTCF binding (Fig. S4D and S4E). However, the expression of the genes adjacent to these TAD boundaries was not significantly changed. These results revealed that the reduced CTCF binding after K20R mutation had little effect on TAD boundaries and the expression of their nearby genes during EB differentiation.

### CTCF regulates the expression of cardiac mesoderm differentiation-associated genes by facilitating enhancer-promoter interactions

CTCF regulates gene expression by mediating the formation of chromatin loops (Zheng and Xie 2019). To explore the relationships among abnormal gene expression, the change in CTCF binding, and its mediated loops, we obtained 21,579 loops and found that most of these loops were CTCF-mediated loops (Fig. 6C). Differential loops consisted of 3940 downregulated and 2566 upregulated loops (Fig. 6D), and were verified with aggregate peak analysis (APA) (Fig. 6E). Classification of the differential loops showed that most were CTCF-mediated loops (Fig. 6F). CTCF could regulate enhancer-promoter interactions (EP interactions) through two different ways either promoting EP interactions by functioning as a structural protein or inhibiting EP interactions by acting as a chromatin insulator (Song et al. 2022). We first isolated EP interactions from the changed CTCF-mediated loops, in which CTCF bound enhancers and promoters, to examine whether CTCF-K20R mutation affects these two different functions of CTCF during the EB differentiation of mESCs. The analysis of the expression of genes associated with these CTCF-mediated EP interactions indicated that only a small fraction of genes showed significant changes in expression, of which most were expressed at low levels (Fig. 6G). Furthermore, the significantly changed and relatively highly expressed genes *Eomes* and *Apex1* were chosen for visual inspection. The tracks showed that the EP interactions and their expression levels were indeed significantly reduced, consistent with the decrease in CTCF binding (Fig. 6H and Fig. S5A). We further analyzed the altered EP interactions and found that the expression of most of the genes associated with the changes in EP interactions was unchanged (Fig. S5B). Moreover, we inspected the expression of the altered genes with changes in EP interactions and found that the change in EP interactions for these genes was not caused by the CTCF insulator function (Fig. S5C). Therefore, CTCF mainly regulates the expression of genes involved in heart development by directly binding to their promoters and enhancers. Taken together, these data suggest that lysine 20 of CTCF might be essential for the early cardiac mesoderm differentiation of mESCs.

## Discussion

CTCF regulates gene activation or repression, chromatin insulation, and distant chromatin interaction by binding to a wide range of sequences via 11 zinc fingers (Ong and Corces 2014). Moreover, CTCF modifications, such as phosphorylation, SUMOylation, and poly(ADP)ribosylation, promote CTCF participation in various biological processes (Docquier et al. 2009, Klenova et al. 2001, MacPherson et al. 2009, Yu et al. 2004). Nevertheless, CTCF acetylation and its related biological functions remain unclear. In the present study, we added another layer of CTCF modification by showing that CTCF is acetylated at lysine 20 by CBP and deacetylated by HDAC6; however, other sites in CTCF may also be acetylated.

Our data indicated that CTCF-K20R mutation regulates the early cardiac differentiation of mESCs, which was similar to the data obtained upon *Ctcf* loss of function. And in vivo* Ctcf* knockout in cardiac progenitor cells during development causes immature mitochondrial development and severe cardiac defects (Gomez-Velazquez et al. 2017). CTCF-K20R mutant selectively reduced CTCF occupancy at a subset of CTCF binding sites, and some of these loci were located at promoters of the genes. The reduced CTCF binding resulted in decreased chromatin accessibility, weakened EP interactions, and caused abnormal expression of genes associated with cardiac development, indicating that CTCF had a direct role in regulating the expression of myocardial differentiation-related genes.

Most biological functions of CTCF are associated with its ability to bind DNA via its 11 zinc fingers; however, the biological functions of the N-terminal domains of CTCF remain poorly understood. The N-terminus of CTCF has an unstructured organization in vitro except in *Drosophila melanogaster*, and in most species, the N-terminal domains of CTCF form dimers, as determined through yeast two-hybrid and coimmunoprecipitation assays (Bonchuk et al. 2020). Furthermore, the N-terminus of CTCF has been shown to interact with cohesin subunit SA2 and mediate chromatin looping (Li et al. 2020, Pugacheva et al. 2020), suggesting that the N-terminal domain of CTCF plays an essential role in genome organization. Indeed, although the CTCF short isoform (CTCF-s), which lacks the N-terminus and 2.5 zinc fingers, competes with canonical CTCF for genomic DNA binding and disrupts the CTCF/cohesin-mediated long-range chromatin loops (Li et al. 2019), CTCF-s could not mediate long-range DNA–DNA interactions and might not regulate cohesin-mediated loop extrusion.

Protein acetylation may mediate the protein interaction, and the CTCF-K20R mutation reduced the interaction between CTCF and CBP, suggesting that lysine 20 acetylation of CTCF might regulate dimer formation and subsequently modulate the organization of CTCF-mediated long-distance interactions. Lysine 20 is present in the N-terminus of CTCF, and additional experiments are needed to determine whether lysine 20 acetylation in CTCF regulates cohesin-mediated loop extrusion.

## Conclusions

In summary, we found that CTCF could be acetylated at K20 by CBP and deacetylated by HDAC6. CTCF-K20R mutation had no effect on mESC self-renewal but hindered cardiac mesoderm differentiation. Mechanism studies revealed that CTCF-K20R mutation resulted in the decrease of a subset of CTCF binding sites during differentiation, further leading to the down-regulation of chromatin accessibility and EP interactions, which both might be harmful to the activation of genes related to cardiac mesoderm differentiation.

## Methods

### Cell culture and differentiation

HEK293T and HeLa cells were cultured in Dulbecco's modified Eagle medium (DMEM) supplemented with 10% FBS and 1% penicillin and streptomycin. The mESC line E14Tg2A was cultured on mitomycin C-inactivated mouse embryo fibroblasts in DMEM containing 15% fetal bovine serum (FBS; Gibco), 1 mM sodium pyruvate (Gibco), 1 mM nonessential amino acids (Gibco), 1 mM GlutaMAX (Gibco), 0.1 mM 2-mercaptoethanol (Gibco), 1000 U/mL leukemia inhibitory factor, and 2i inhibitors (3 μM CHIR99021 and 1 μM PD0325901). All mammalian cell lines were grown in a 37 °C incubator with 5% CO_2_. For ESC differentiation, ESCs were differentiated into EBs using the hanging-drop method (with 1,000 cells/drop) in DMEM supplemented with 15% serum without LIF.

### Plasmid construction

shRNA oligos were designed and cloned into the pLKO.1 plasmid. The sequences of shRNA oligos are listed in Supplementary Table 1. The *CTCF* or *CTCF-K20R* cDNAs were cloned into pSin-FLAG and pGEX4T-2 vectors, and the sequences of their primers are listed in Supplementary Table 2.

### Lentivirus-mediated shRNA knockdown

The recombinant constructs (pLKO.1 empty, pLKO.1-*CTCF*, pLKO.1-*HDAC6,* and pLKO.1-*MOF*) were transfected into HEK293T cells together with lentiviral helper plasmids (pMD2.G and psPAX.2). Viral supernatants were collected after 48 h, filtered through a 0.45 μm filter and then mixed with 8 μg/mL polybrene to infect the target cells. Stable cell lines were selected with 2 μg/mL puromycin 2 days after infection.

### Generation of CTCF-K20R mESCs

A point mutation in CTCF in which lysine 20 was replaced with arginine (K20R) was generated in mouse ESCs using the CRISPR/Cas9 system. The sgRNA target sequences were designed using crispr.mit.edu and inserted into the pX330 plasmid. Then, pX330, along with the linearized donor vector, were electroporated into mESCs for gene editing. The correctly targeted colonies were chosen through drug selection, genomic DNA sequencing, and Western blotting. The oligos used for generating mESCs expressing the CTCF-K20R point mutation are shown in Supplementary Table 3.

### Antibodies

The following antibodies were used in this study: rabbit anti-CTCF (active motif, 61,311), mouse anti-Flag (Sigma, F1804), rabbit anti-acetylated lysine (Cell Signaling Technology, 9441S), mouse anti-β-ACTIN (Abcam, ab8226), rabbit anti-HDAC6 (Proteintech, 12,834–1-AP), rabbit anti-CBP (Cell Signaling Technology, 7389S), mouse anti-MOF (Boster, A02757), rabbit anti-H3K27ac (Active Motif, 39,133), anti-OCT4 (Santa Cruz Biotechnology, sc-5279), anti-SOX2 (Abcam, ab79351), mouse anti-cTnT (Thermo, MA5-12,960), rabbit IgG (Santa Cruz Biotechnology, sc-2027), and anti-6 × HIS (Abcam, ab18184).

### Immunoprecipitation and Western blotting

Cells were harvested and lysed in cell lysis buffer (50 mM Tris–HCl (pH 7.6), 1% Triton X-100, 1 mM EDTA, 10% glycerol, 1 mM DTT, 1 mM PMSF, and protease inhibitor cocktail). Proteins were resolved on an SDS–PAGE gel and then transferred to a PVDF membrane. Then, the membrane was washed with TBS-T buffer, and immunoblotting was performed with the indicated antibodies.

For immunoprecipitation, cells were lysed in lysis buffer (20 mM Tris–HCl (pH 7.5), 1% Triton X-100, 150 mM NaCl, and 10% glycerol) supplemented with a complete protease inhibitor cocktail. Then, immunoprecipitation was performed using the indicated antibodies. Generally, 2 μg of antibody was added to Protein A/G beads and incubated at 4 °C for 6 h before the supernatant was discarded. After adding the protein extract, the incubation was continued for 12 h, and then immune complexes were washed four times with lysis buffer, resolved on SDS–PAGE gels, and analyzed using immunoblotting. Western blot results were quantified with ImageJ software.

### Dot blot

CTCF-K20 acetylated and unmodified polypeptides were synthesized by Guangzhou IGE Biotechnology. Equal amounts of polypeptides containing K20 acetylated polypeptide and unmodified polypeptide were incubated with an equal amount of diluted anti-CTCF-K20Ac antibody at 37 °C. The immunoblot analysis was performed with anti-CTCF-K20Ac antibodies.

### Quantitative RT–PCR analysis

Total RNA was isolated from samples with TRIzol reagent (Invitrogen). One milligram of total RNA was then reverse transcribed with a reverse transcription system (Promega). The cDNAs of interest were then quantified using real-time qPCR. The primers used in the RT–qPCR assays are listed in Supplementary Table 4. All experiments were repeated three times.

### RNA sequencing and bioinformatics analysis

Total RNA was extracted as described above. RNA sequencing libraries were constructed using the VAHTS mRNA-seq V3 Library Prep Kit (Vazyme Biotech, NR611). Two rounds of mRNA purification were performed to guarantee the removal of rRNA. Briefly, 50 μL of mRNA capture beads were incubated with 1.5 μg of total RNA at 65 °C for 5 min and then at 25 °C for 5 min. The supernatant was discarded, and 200 μL of bead wash buffer were added to clean the beads. Fifty microliters of Tris buffer were added to resuspend the beads, and the sample was incubated at 80 °C for 5 min to release mRNA. Then, 50 μL of bead binding buffer were added to facilitate the binding of mRNA to the beads. Furthermore, ribosome-depleted mRNAs were fragmented at 85 °C for 6 min, and cDNAs were synthesized. The cDNAs were purified with AMPure XP beads (Beckman Coulter, A63882), followed by end repair, adaptor ligation, size selection of the library, and library amplification. The libraries from two biological replicates were purified using AMPure XP beads and then sequenced with an illumina NovaSeq instrument (Annoroad Gene Technology Co., Ltd.).

Adaptors were trimmed from raw reads using Trim_Galore (v0.4.4) and then quasi-mapped and quantified to the mouse mm10 genome with salmon (v0.9.1) (Patro et al. 2017). Transcript-level counts were collapsed to gene-level counts using tximport (v1.20.0) (Soneson et al. 2015) and analyzed with DESeq2 (v1.10.1) (Love et al. 2014). Genes with a fold change greater than 2 and a q-value less than 0.05 were considered differentially expressed genes (DEGs). The DEGs are listed in Supplementary Table 5. GO analysis and GSEA were conducted with clusterProfiler (v4.0.0) (Yu et al. 2012).

### GST pull-down assay

BL21 *E. coli* were treated with 0.1 mM IPTG at 37 °C to induce protein expression, harvested, and resuspended in PBS containing 0.5% Triton X-100, 2 mM EDTA, and 1 mM PMSF, followed by sonication. The protein was then purified using GST antibody-conjugated beads. Beads were subsequently added to HEK293T cell lysate transfected with the HDAC6-Flag construct and mixed at 4 °C overnight. Next, beads were harvested through centrifugation and washed four times with 0.3% Nonidet P40 buffer before boiling with 1 × SDS–PAGE loading buffer and subjected to Western blotting.

### Cellular immunofluorescence staining

Cells were fixed with 4% paraformaldehyde and permeabilized with 0.2% Triton X-100 containing 10% FBS (Invitrogen)/1% BSA in PBS at room temperature for 15 min. Samples were then incubated with primary antibodies at 4 °C overnight. The antibody used for cellular immunofluorescence staining was raised against cTnT. The cells were then washed four times, and 0.1 μg/mL DAPI (Sigma) was included in the final wash to stain nuclei. Images were captured with an inverted microscope (DMI4000, Leica Microsystems).

### Flow cytometry

mESCs were dissociated with trypsin, and cells were fixed with 4% paraformaldehyde and permeabilized with 0.2% Triton X-100 containing 10% FBS (Invitrogen)/1% BSA in PBS at room temperature for 15 min. Samples were then incubated with primary antibodies at 37 °C for 30 min. Cells were washed twice and incubated with a secondary antibody in a cassette at room temperature for 30 min. The cTnT antibody was used in this experiment. The analysis was performed using FlowJo software.

### Chromatin immunoprecipitation and sequencing

ChIP experiments were performed as previously described (Li et al. 2019). Briefly, 1 × 10^7^ cells were crosslinked with 1% formaldehyde at room temperature for 10 min. Then, the crosslinking reaction was stopped by adding glycine (final concentration, 0.125 M). The cells were sonicated in SDS lysis buffer containing a 1 × protease inhibitor cocktail and 1 mM PMSF to achieve a chromatin size of 100–300 bp. The sonicated chromatin was incubated with the indicated antibodies coupled with Dynabeads conjugated to Protein A and G (1:1 mixed) at 4 °C overnight with rotation. Immune complexes were washed with the following buffers: low-salt wash buffer (0.1% SDS, 1% Triton X-100, 2 mM EDTA, 20 mM Tris–HCl (pH 8.0), and 150 mM NaCl), high-salt wash buffer (0.1% SDS, 1% Triton X-100, 2 mM EDTA, 20 mM Tris–HCl (pH 8.0), and 500 mM NaCl), LiCl wash buffer (0.25 M LiCl, 1% IGEPAL CA-630, 1% deoxycholic acid (sodium salt), 1 mM EDTA, and 10 mM Tris–HCl (pH 8.0)) and TE buffer (10 mM Tris–HCl (pH 8.0) and 1 mM EDTA). After reversing the crosslinks, the ChIPed DNA samples were purified, and libraries were constructed according to the Illumina ChIP-seq library generation protocol.

### ATAC sequencing

ATAC-seq experiments were performed as previously described (Buenrostro et al. 2015). Briefly, 50,000 cells were harvested and washed once with 50 μL of cold PBS. Then, the cells were resuspended in 50 μL of lysis buffer (10 mM Tris–HCl (pH 7.4), 10 mM NaCl, 3 mM MgCl_2_, and 0.2% (v/v) IGEPAL CA-630). Then, the suspension of nuclei was centrifuged at 500 × g at 4 °C for 10 min. The pellet was resuspended in 50 μL of transposition reaction mix (10 μL of TD buffer, 5 μL of Tn5 transposase, and 35 μL of nuclease-free H_2_O) and incubated at 37 °C for 30 min. Finally, DNA was extracted using a MinElute PCR Purification Kit (QIAGEN). ATAC-seq libraries were constructed and purified with AMPure XP beads (Beckman Coulter). The libraries were denatured and diluted and then sequenced with the HiSeq X-Ten platform (Annoroad Gene Technology Co., Ltd).

### ATAC-seq and ChIP-seq data analysis

All ATAC-seq and CTCF ChIP-seq experiments were performed using two biological replicates. Raw reads were trimmed with Trim_Galore and aligned to the mouse mm10 genome using bowtie2 (v2.2.5) (Langmead and Salzberg 2012) with the parameter “–very-sensitive –end-to-end –no-unal”. Reads with a mapping quality lower than 30 were discarded. Duplicate reads were removed with sambamba (v0.6.7). Reads overlapping with mouse mm10 blacklist regions (http://mitra.stanford.edu/kundaje/akundaje/release/blacklists) were excluded. For the CTCF ChIP-seq experiment, 10 million reads were randomly subsampled. Peaks were called using MACS2 (v2.1.0) (Zhang et al. 2008) with default parameters for CTCF ChIP-seq and ATAC-seq experiments. Broad peaks were called for histone H3K27ac ChIP-seq using MACS2 with the parameter “–broad”. CTCF peak summits were submitted to the Homer findMotifsGenome.pl tool (Heinz et al. 2010) for the motif analysis with the parameters “-len 8,10,12,20”. bamCoverage from deepTools (v2.2.4) (Ramirez et al. 2016) was used to generate a normalized bigwig file with the parameter “-of bigwig -bs 1 –normalizeUsing RPGC”. Histone H3K27ac bigwig files were normalized such that the total enrichment of all H3K27ac peaks was similar. Regions that were enriched with H3K27ac and overlapped with chromatin-accessible peaks but were not transcription start sites (TSSs) or transcription termination sites (TTSs) were identified as enhancers. Heatmaps were drawn with deepTools (v2.2.4). Differential sites were identified using DiffBind (v3.2.4) (Ross-Innes et al. 2012) with default parameters, and peaks with *q* values less than 0.05 and log_2_(fold change) values greater than 1 were considered differential binding sites. The analysis of differential CTCF binding in day 4 differentiated cells is provided in Supplementary Table 6.

### BL-Hi-C experiments

The BL-Hi-C libraries were constructed as previously described (Liang et al. 2017). Briefly, the cells were treated with 1% formaldehyde at room temperature for 10 min, and the crosslinking reaction was quenched by adding 2.5 M glycine to a final concentration of 0.2 M. Then, the cells were resuspended with BL-Hi-C lysis buffer 1 and incubated on ice for 15 min. After centrifugation, the cell pellet was resuspended in BL-Hi-C lysis buffer 2 and rotated at 4 °C for 15 min. The cell pellet was washed once with BL-Hi-C lysis buffer 1, resuspended with 0.5% SDS and incubated at 62 °C. At the end of the incubation, SDS was quenched by adding 10% Triton X-100 and ddH_2_O, and the sample was incubated at 37 °C for 10 min. Afterward, the genomic DNA was digested with HaeIII (NEB) at 37 °C for 2 h to generate blunt-end fragments. Chromatin was cleaved by adding HaeIII (NEB) and incubating the sample at 37 °C. Cleaved chromatin was A-tailed by adding a 10 mM dATP solution (Thermo) and Klenow Fragment (NEB) with rotation at 37 °C for 40 min. Then, chromatin was treated with adenine and ligated with biotinylated bridge linker S2 (annealed by /5Phos/CGCGATATC/iBIOdT/TATCTGACT and /5Phos/GTCAGATAAGATATCGCGT) at 16 °C for 4 h. The unligated DNA fragments were digested with DNA exonuclease (NEB) at 37 °C for 1 h. After centrifugation at 3500 g at 4 °C for 5 min and removal of the supernatant, the pellet was resuspended in ddH_2_O with lambda exonuclease buffer, lambda exonuclease, and exonuclease I and rocked at 900 rpm at 37 °C for 1 h in a ThermoMixer C. Next, the samples were treated with SDS and proteinase K at 55 °C overnight to digest the proteins, and the DNA was purified using phenol:chloroform:isoamyl alcohol (25:24:1) extraction followed by ethanol precipitation. Then, the DNA was fragmented into 300 bp fragments on average by sonication, and the biotin-labeled DNA fragments were pulled down with Dynabeads M-280 conjugated to streptavidin. The beads were washed twice with 2 × B&W buffer (10 mM Tris–HCl (pH 7.5), 1 mM EDTA, and 2 M NaCl) and blocked with 1 × I-Block buffer (2% I-block protein-based blocking reagent and 0.5% SDS) at room temperature for 45 min. Next, the beads were washed twice with 1 × B&W buffer and treated with 1 mg of preheated salmon sperm DNA with rotation at room temperature for 30 min. After washing with 1 × B&W buffer, the beads were resuspended with 2 × B&W buffer, combined with sonicated DNA and rotated at room temperature for 45 min. The beads were washed five times with 2 × SSC containing 0.5% SDS, twice with 1 × B&W buffer and once with Buffer EB (QIAGEN). DNA bound to beads was end-repaired using T4 DNA polymerase (NEB), T4 polynucleotide kinase (NEB) and large (Klenow) fragment (NEB) with shaking at 900 rpm at 37 °C for 30 min. After two washes with 1 × TWB (5 mM Tris–HCl (pH 7.5), 0.5 mM EDTA, 1 M NaCl, and 0.05% Tween 20) at 55 °C for 2 min, DNA on beads was A-tailed using Klenow fragment (3’/5’ exo-) (NEB) with shaking at 900 rpm at 37 °C for 30 min. Beads were washed twice with 1 × TWB at 55 °C for 2 min and once with 1 × Quick ligation buffer (NEB). DNA on beads was ligated with an adaptor using Quick ligase (NEB) and 20 mM Y-Adaptor (Annealed by /5Phos/GATCGGAAGAGCACACGTCTGAACTCCAGTCAC and TACACTCTTTCCCTACACGACGCTCTTCCGATCT) at room temperature for 45 min. Beads were washed twice with 1 × TWB at 55 °C for 2 min and once with EB buffer (QIAGEN).

The libraries were constructed using Q5 Hot Star DNA Polymerase (NEB) for PCR amplification. PCR products with sizes ranging from 300–700 bp were purified using Ampure XP beads (Beckman Coulter) and subjected to sequencing with the Illumina NovaSeq platform (Annoroad Gene Technology Co., Ltd.).

### BL-Hi-C analysis

All BL-Hi-C experiments were performed using two biological replicates. Raw reads were first submitted to Trim_Galore to remove adaptors, and then linkers were trimmed with the parameters “-m 1 -k 2 -e 1 -l 15 -A ACGCGATATCTTATC -B AGTCAGATAAGATAT” using ChIA-PET2 (v0.9.3) (Li et al. 2017). The trimmed reads were handled using HiC-Pro (v2.11.1) (Servant et al. 2015). Quality control information was collected and is listed in Supplementary Table 7. The distance decay analysis was performed using hicPlotDistVsCounts from the hicexplorer suite (v3.6) at a 100 kb resolution (Wolff et al. 2020). The reproducibility of Hi-C data was assessed using HiCRep (v0.2.6) for all chromosomes except chrY and chrM at 50 kb resolution (Yang et al. 2017), and the average correlation score for all chromosomes was used. Hi-C data were normalized using HiCcompare (v1.14.0) (Stansfield et al. 2018). The normalized matrix was transformed to Hi-C format using juicer tools (v1.13) (Durand et al. 2016) and visualized in WashU Epigenome Browser (Li et al. 2019). Compartments were analyzed using juicer tools with default parameters at 100 kb resolution, and the insulation score and TAD boundaries were identified by FAN-C (v0.9.20) at 100 kb resolution with parameter “-w 400 kb” (Kruse et al. 2020). Significant loops were identified as previously described (Song et al. 2022) and are listed in Supplementary Table 8.

### Quantification and statistical analysis

Two-tailed Student’s *t* tests were used for all comparisons, including the RT–qPCR analysis. All values included in the figures are presented as the means ± s.d. Error bars represent ± s.d. for triplicate experiments. The statistical significance is indicated with asterisks (*). A two-sided *P* value of < 0.05 was considered to be statistically significant (*P < 0.05, **P < 0.01, and ***P < 0.001).

## Supplementary Information

**Additional file 1.** Supplementary tables

**Additional file 2.** Supplementary movie 1

**Additional file 3.** Supplementary movie 2

**Additional file 4.** Supplementary figures

## Data Availability

The RNA-seq, ATAC-seq, ChIP-seq, and BL-Hi-C data described in this paper are deposited in GEO under accession number GSE181705 and GSA under accession number CRA004748.

## Abbreviations

CTCF: CCCTC-binding factor
CTCF-K20: Lysine 20 of CTCF
mESCs: Mouse embryonic stem cells
TADs: Topologically associated domains
CKII: Casein kinase II
CBP: CREB-binding protein
ChIP-seq: Chromatin immunoprecipitation sequencing
EP interactions: Enhancer-promoter interactions
TSA: Trichostatin A
co-IP: Co-immunoprecipitation
NAM: Nicotinamide
shRNA: Short hairpin RNA
TBSA: Tubastatin A
CRISPR: Clustered regularly interspaced short palindromic repeats
EBs: Embryoid bodies
WT: Wild-type
GO: Gene Ontology
GSEA: Gene set enrichment analysis
APA: Aggregate peak analysis
