# Supplementary material for: CTCF acetylation at lysine 20 is required for the early cardiac mesoderm differentiation of embryonic stem cells
Source: Cell Regen. 2022 Sep 19;11:34. doi: 10.1186/s13619-022-00131-w (PMC9482892; doi:10.1186/s13619-022-00131-w)

Fig. 1

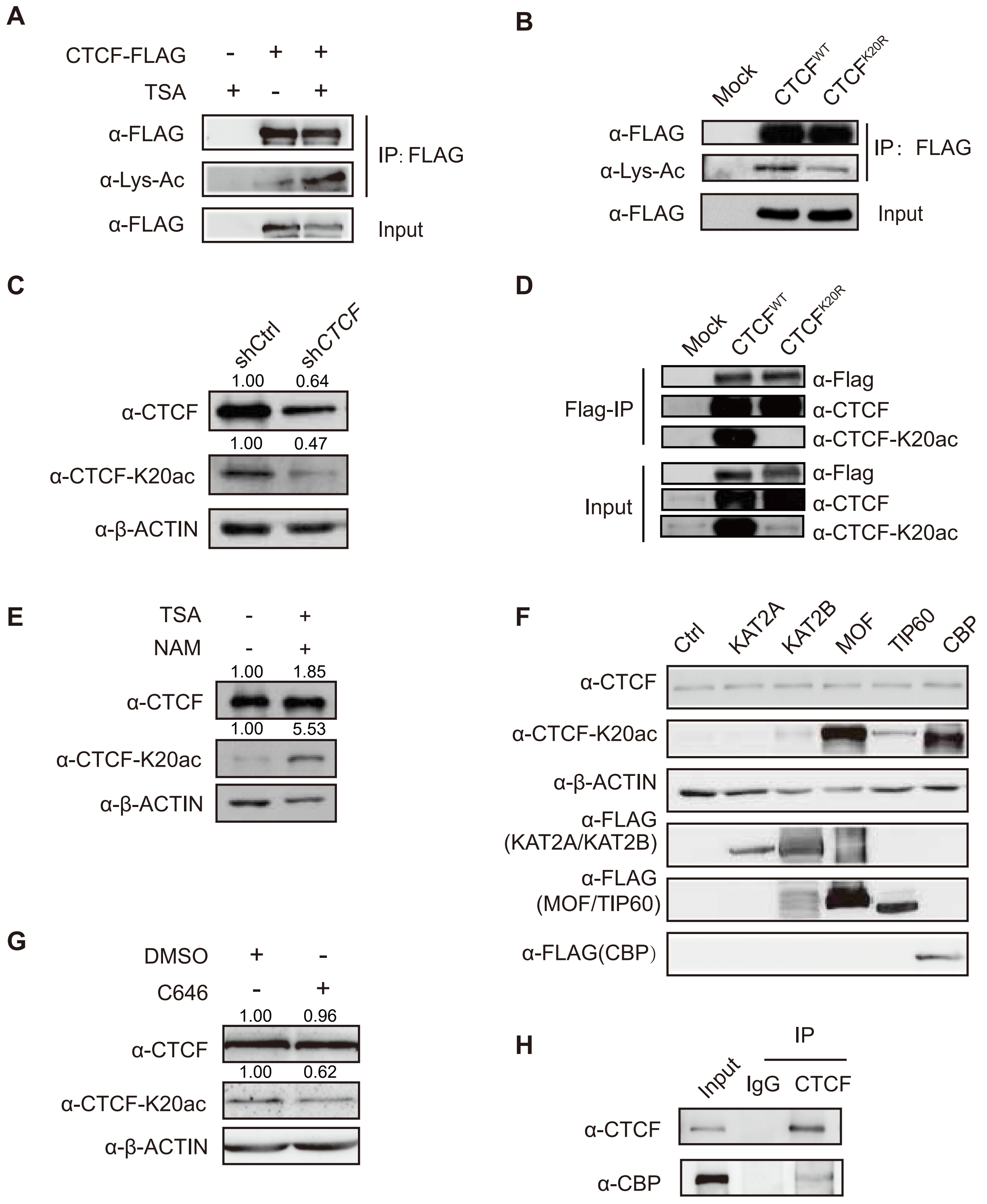

A
CTCF-FLAG - + +
TSA + - +
α-FLAG
α-Lys-Ac
IP: FLAG
α-FLAG
Input
B
Mock
CTCF^WT
CTCF^K20R
α-FLAG
α-Lys-Ac
IP: FLAG
α-FLAG
Input
C
shCtrl
shCTCF
1.00 0.64
α-CTCF
1.00 0.47
α-CTCF-K20ac
α-β-ACTIN
D
Mock
CTCF^WT
CTCF^K20R
Flag-IP
α-Flag
α-CTCF
α-CTCF-K20ac
Input
α-Flag
α-CTCF
α-CTCF-K20ac
E
TSA - +
NAM - +
1.00 1.85
α-CTCF
1.00 5.53
α-CTCF-K20ac
α-β-ACTIN
F
Ctrl
KAT2A
KAT2B
MOF
TIP60
CBP
α-CTCF
α-CTCF-K20ac
α-β-ACTIN
α-FLAG (KAT2A/KAT2B)
α-FLAG (MOF/TIP60)
α-FLAG(CBP)
G
DMSO + -
C646 - +
1.00 0.96
α-CTCF
1.00 0.62
α-CTCF-K20ac
α-β-ACTIN
H
IP
Input
IgG
CTCF
α-CTCF
α-CBP

Fig. 2

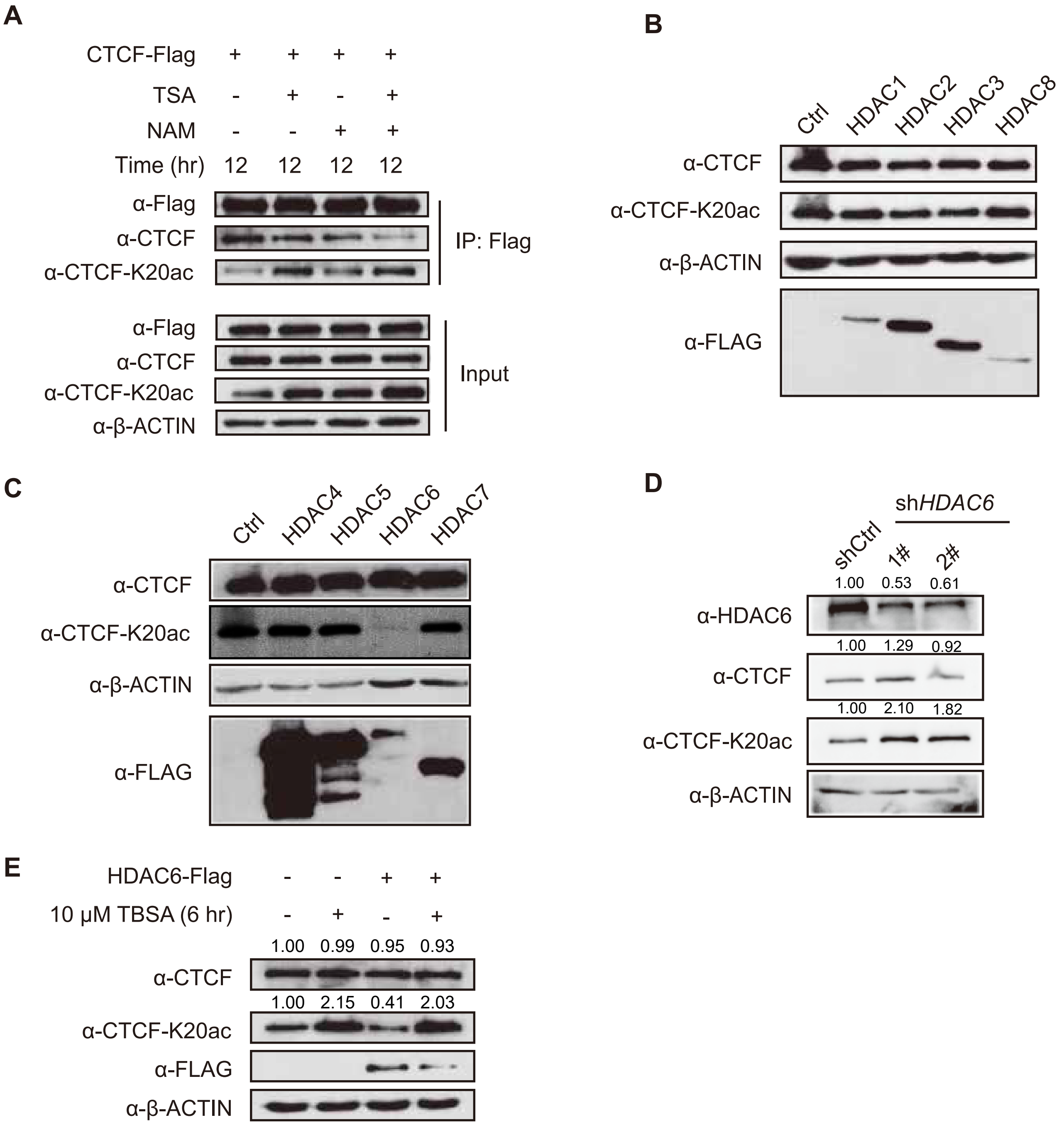
A
CTCF-Flag + + + +
TSA - + - +
NAM - - + +
Time (hr) 12 12 12 12
α-Flag
α-CTCF
α-CTCF-K20ac
IP: Flag
α-Flag
α-CTCF
α-CTCF-K20ac
α-β-ACTIN
Input
B
Ctrl HDAC1 HDAC2 HDAC3 HDAC8
α-CTCF
α-CTCF-K20ac
α-β-ACTIN
α-FLAG
C
Ctrl HDAC4 HDAC5 HDAC6 HDAC7
α-CTCF
α-CTCF-K20ac
α-β-ACTIN
α-FLAG
D
shHDAC6
shCtrl 1# 2#
1.00 0.53 0.61
α-HDAC6
1.00 1.29 0.92
α-CTCF
1.00 2.10 1.82
α-CTCF-K20ac
α-β-ACTIN
E
HDAC6-Flag - - + +
10 μM TBSA (6 hr) - + - +
1.00 0.99 0.95 0.93
α-CTCF
1.00 2.15 0.41 2.03
α-CTCF-K20ac
α-FLAG
α-β-ACTIN

Fig. 3

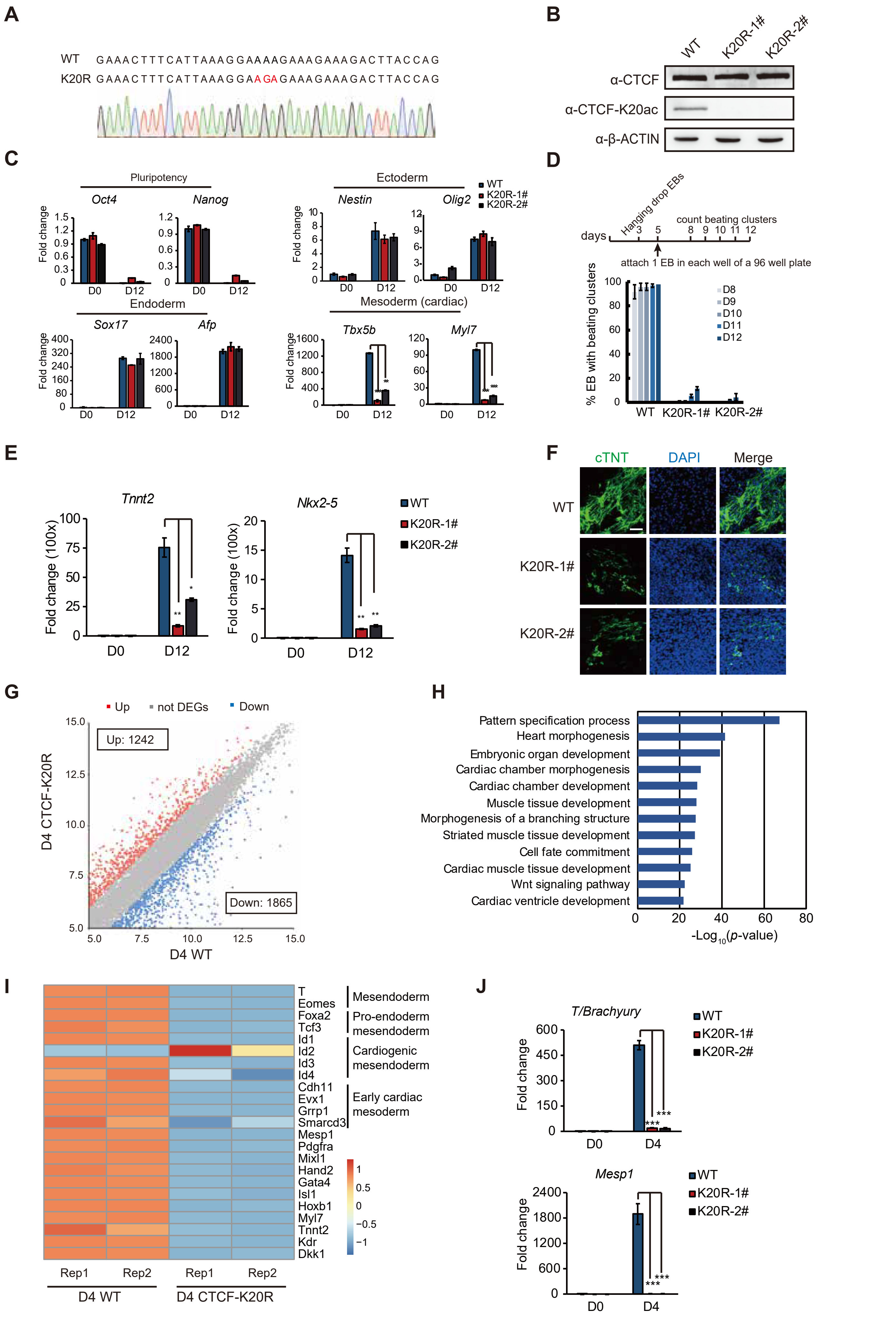
A
WT GAAACTTTCATTAAAGGAAAAGAAAGAAAGACTTACCAG
K20R GAAACTTTCATTAAAGGAAGAGAAAGAAAGACTTACCAG
B
WT
K20R-1#
K20R-2#
α-CTCF
α-CTCF-K20ac
α-β-ACTIN
C
Pluripotency
Oct4
Nanog
Ectoderm
Nestin
Olig2
WT
K20R-1#
K20R-2#
Fold change
D0
D12
Endoderm
Sox17
Afp
Mesoderm (cardiac)
Tbx5b
Myl7
D
days
Hanging drop EBs
3
5
count beating clusters
8 9 10 11 12
attach 1 EB in each well of a 96 well plate
% EB with beating clusters
D8
D9
D10
D11
D12
WT
K20R-1#
K20R-2#
E
Tnnt2
Nkx2-5
Fold change (100x)
WT
K20R-1#
K20R-2#
D0
D12
F
cTNT
DAPI
Merge
WT
K20R-1#
K20R-2#
G
Up
not DEGs
Down
Up: 1242
Down: 1865
D4 CTCF-K20R
D4 WT
H
Pattern specification process
Heart morphogenesis
Embryonic organ development
Cardiac chamber morphogenesis
Cardiac chamber development
Muscle tissue development
Morphogenesis of a branching structure
Striated muscle tissue development
Cell fate commitment
Cardiac muscle tissue development
Wnt signaling pathway
Cardiac ventricle development
-Log10(p-value)
I
T
Eomes
Foxa2
Tcf3
Id1
Id2
Id3
Id4
Cdh11
Evx1
Grrp1
Smarcd3
Mesp1
Pdgfra
Mixl1
Hand2
Gata4
Isl1
Hoxb1
Myl7
Tnnt2
Kdr
Dkk1
Mesendoderm
Pro-endoderm mesendoderm
Cardiogenic mesendoderm
Early cardiac mesoderm
Rep1
Rep2
Rep1
Rep2
D4 WT
D4 CTCF-K20R
J
T/Brachyury
Mesp1
Fold change
WT
K20R-1#
K20R-2#
D0
D4

Fig. 4

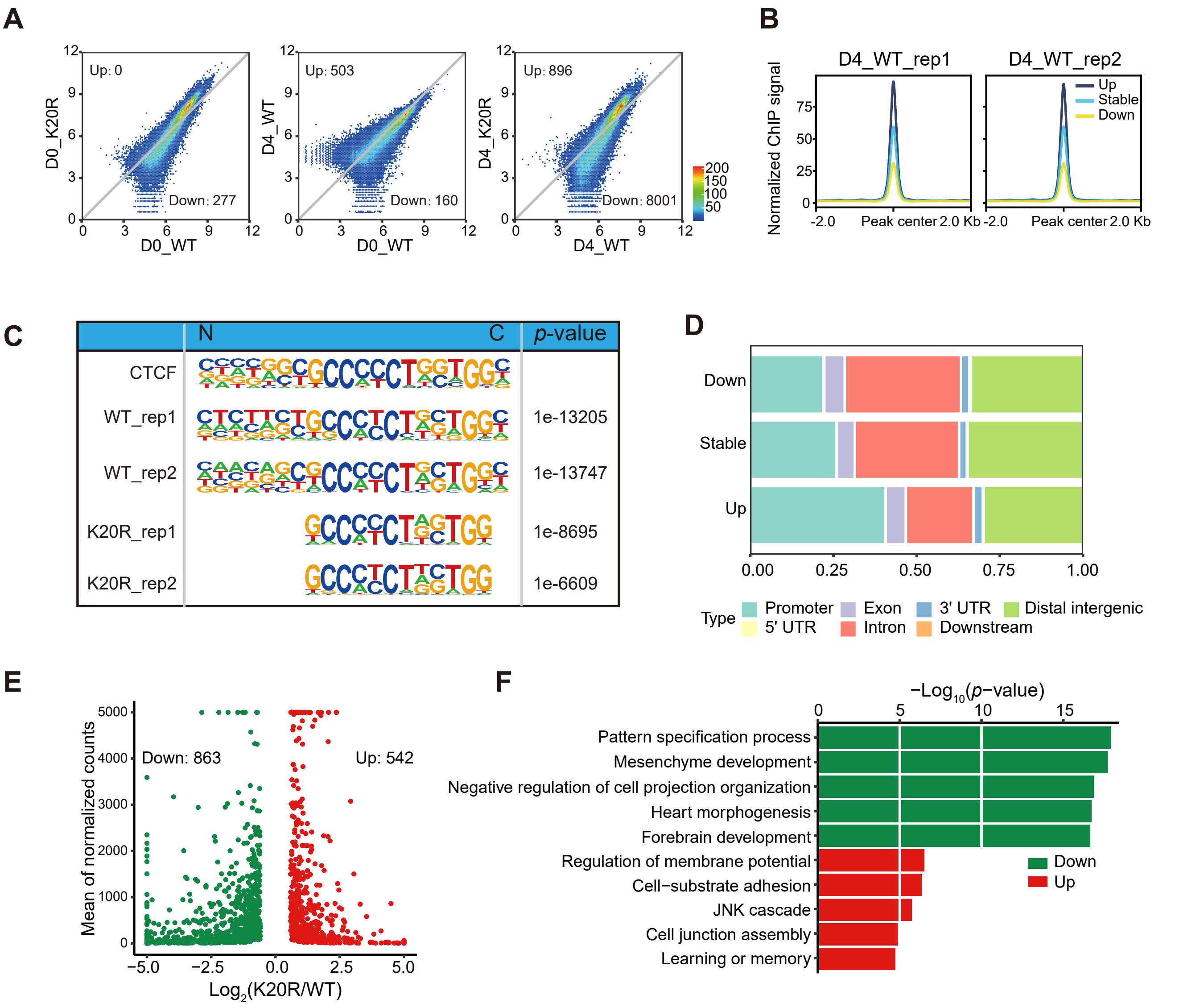
A
Up: 0
Down: 277
D0_K20R
D0_WT
Up: 503
Down: 160
D4_WT
D0_WT
Up: 896
Down: 8001
D4_K20R
D4_WT
200
150
100
50
B
D4_WT_rep1
D4_WT_rep2
Normalized ChIP signal
Up
Stable
Down
-2.0
Peak center
2.0 Kb
C
N
C
p-value
CTCF
WT_rep1
1e-13205
WT_rep2
1e-13747
K20R_rep1
1e-8695
K20R_rep2
1e-6609
D
Down
Stable
Up
0.00
0.25
0.50
0.75
1.00
Type
Promoter
Exon
3' UTR
Distal intergenic
5' UTR
Intron
Downstream
E
Mean of normalized counts
Down: 863
Up: 542
Log2(K20R/WT)
F
−Log10(p−value)
Pattern specification process
Mesenchyme development
Negative regulation of cell projection organization
Heart morphogenesis
Forebrain development
Regulation of membrane potential
Cell−substrate adhesion
JNK cascade
Cell junction assembly
Learning or memory
Down
Up

Fig. 5

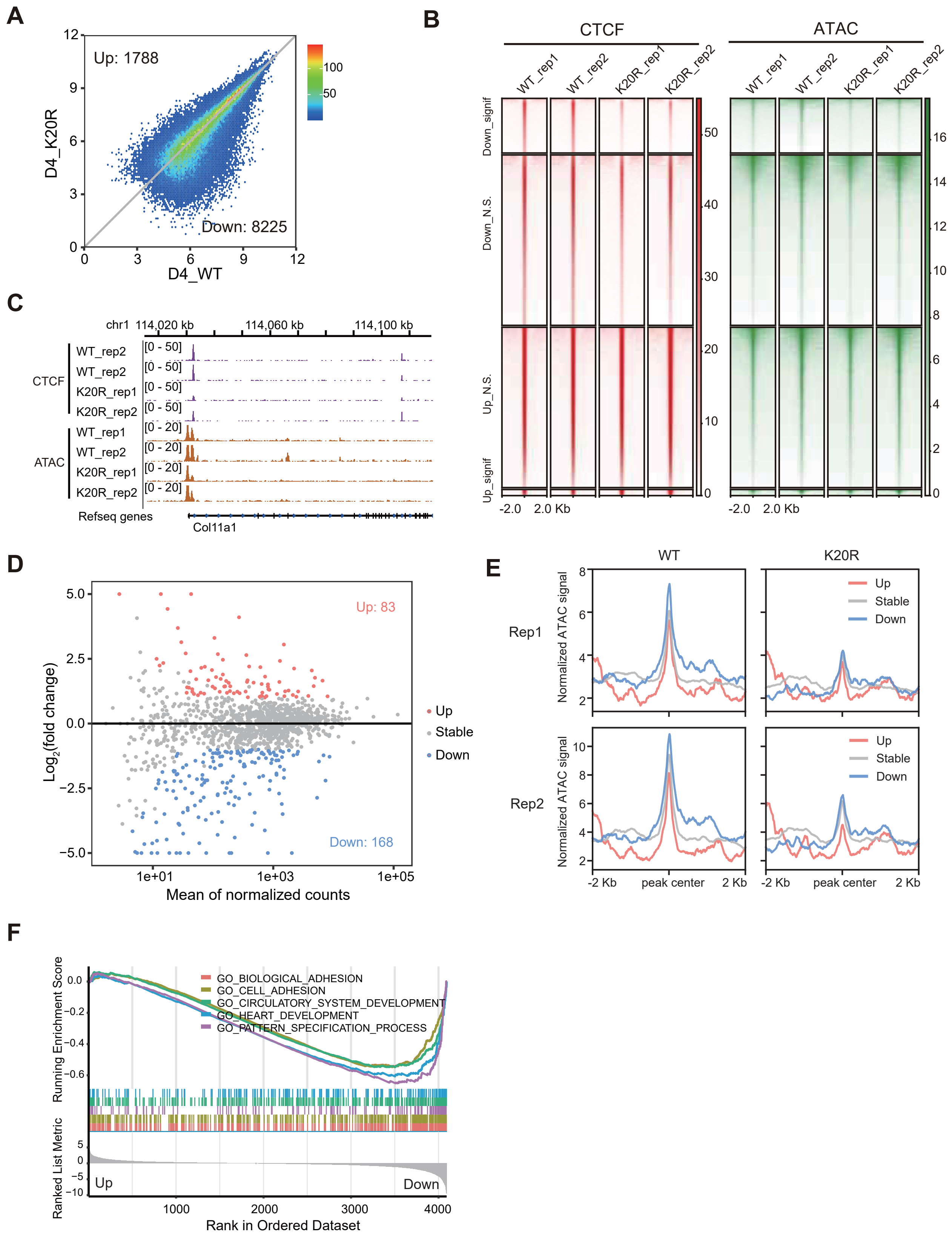

A
Up: 1788
Down: 8225
D4_K20R
D4_WT
100
50
B
CTCF
ATAC
WT_rep1
WT_rep2
K20R_rep1
K20R_rep2
Down_signif
Down_N.S.
Up_N.S.
Up_signif
-2.0
2.0 Kb
C
chr1
114,020 kb
114,060 kb
114,100 kb
CTCF
ATAC
[0 - 50]
[0 - 20]
Refseq genes
Col11a1
D
Up: 83
Down: 168
Log2(fold change)
Mean of normalized counts
Up
Stable
Down
E
WT
K20R
Rep1
Rep2
Normalized ATAC signal
-2 Kb
peak center
2 Kb
F
GO_BIOLOGICAL_ADHESION
GO_CELL_ADHESION
GO_CIRCULATORY_SYSTEM_DEVELOPMENT
GO_HEART_DEVELOPMENT
GO_PATTERN_SPECIFICATION_PROCESS
Running Enrichment Score
Ranked List Metric
Rank in Ordered Dataset
Up
Down

Fig. 6

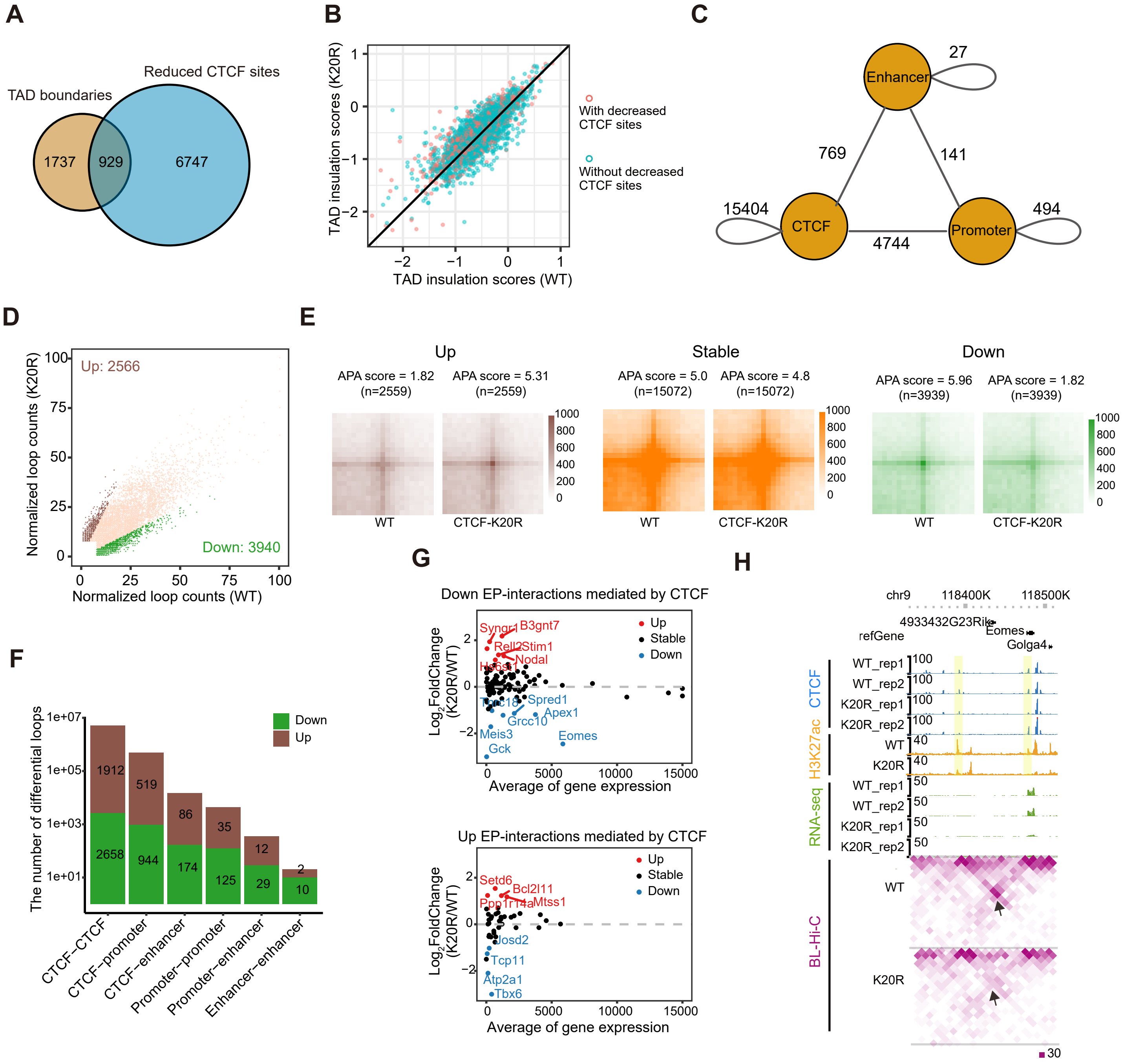
A
TAD boundaries
Reduced CTCF sites
1737
929
6747
B
TAD insulation scores (K20R)
TAD insulation scores (WT)
With decreased CTCF sites
Without decreased CTCF sites
C
Enhancer
CTCF
Promoter
27
769
141
15404
4744
494
D
Normalized loop counts (K20R)
Normalized loop counts (WT)
Up: 2566
Down: 3940
E
Up
Stable
Down
APA score = 1.82 (n=2559)
APA score = 5.31 (n=2559)
APA score = 5.0 (n=15072)
APA score = 4.8 (n=15072)
APA score = 5.96 (n=3939)
APA score = 1.82 (n=3939)
WT
CTCF-K20R
F
The number of differential loops
Down
Up
1912
519
86
35
12
2
2658
944
174
125
29
10
CTCF–CTCF
CTCF–promoter
CTCF–enhancer
Promoter–promoter
Promoter–enhancer
Enhancer–enhancer
G
Down EP-interactions mediated by CTCF
Up EP-interactions mediated by CTCF
Log2FoldChange (K20R/WT)
Average of gene expression
Up
Stable
Down
Syngr1
B3gnt7
Rell2
Stim1
Nodal
Spred1
Apex1
Grcc10
Meis3
Eomes
Gck
Setd6
Bcl2l11
Ppp1r14a
Mtss1
Josd2
Tcp11
Atp2a1
Tbx6
H
chr9
118400K
118500K
4933432G23Rik
Eomes
Golga4
refGene
CTCF
H3K27ac
RNA-seq
BL-Hi-C
WT_rep1
WT_rep2
K20R_rep1
K20R_rep2
WT
K20R
30

Supplementary Fig. 1

A

H-CTCF 11 EESETFIKG K ERKTYQRRRE 30
M-CTCF 11 EESETFIKG K ERKTYQRRRE 30
C-CTCF 11 EESETFIKG K ERKTYQRRRE 30
R-CTCF 11 EESETFIKG K ERKTYQRRRE 30
Z-CTCF 11 EESETFIKG K ERKTYQRRRE 30

B

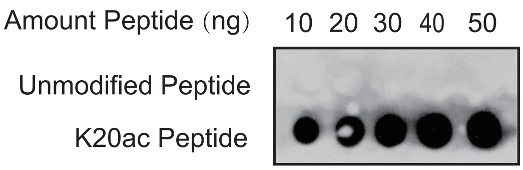

C

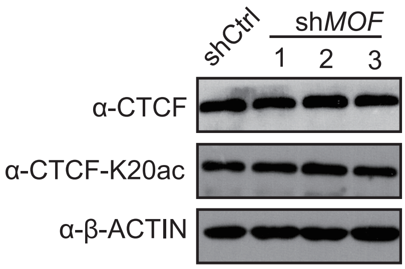

D

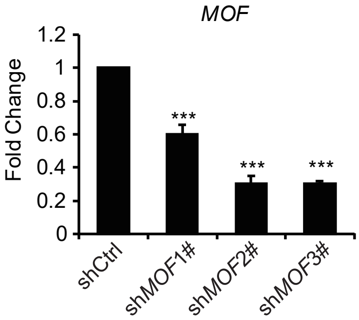

Supplementary Fig. 2

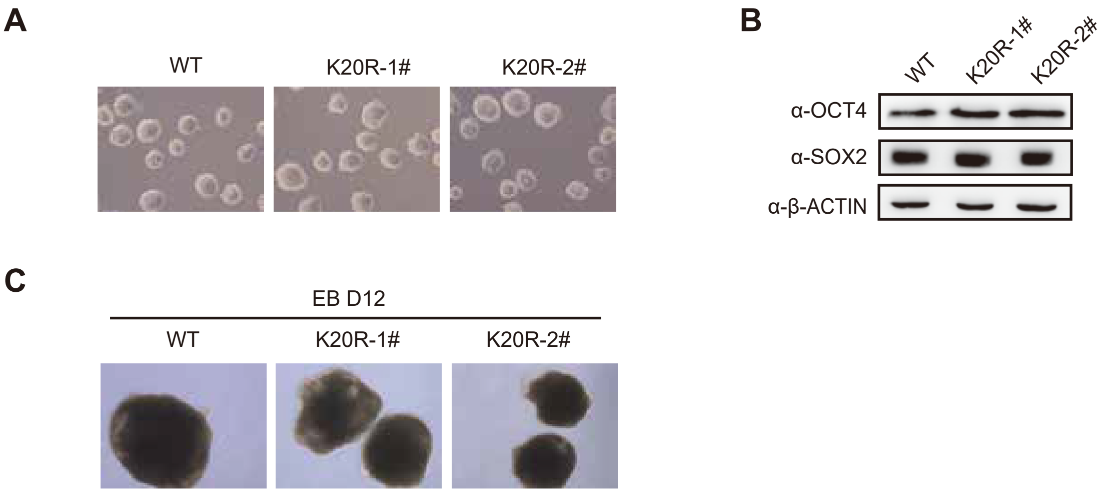

Supplementary Fig. 3

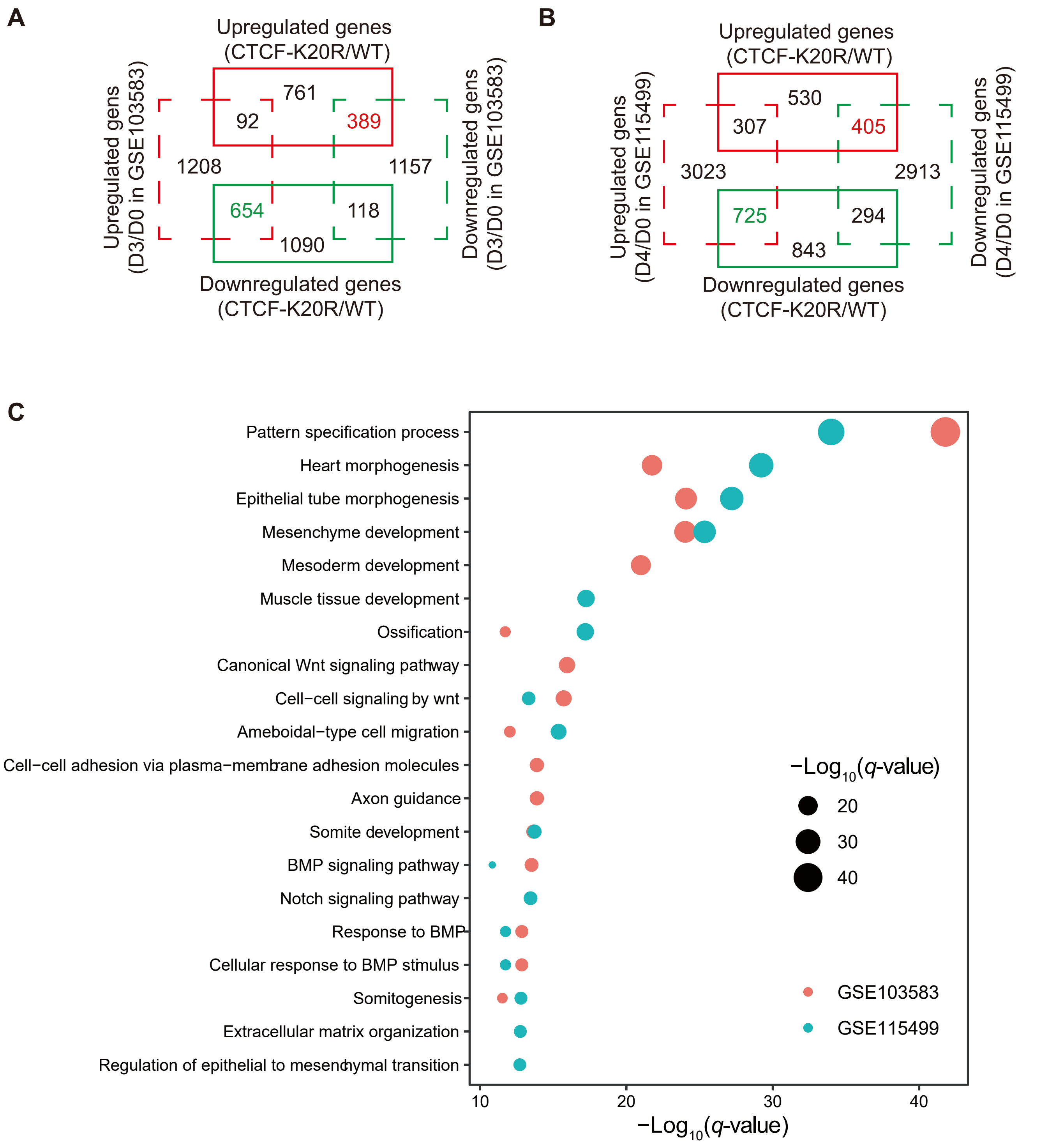

Supplementary Fig. 4

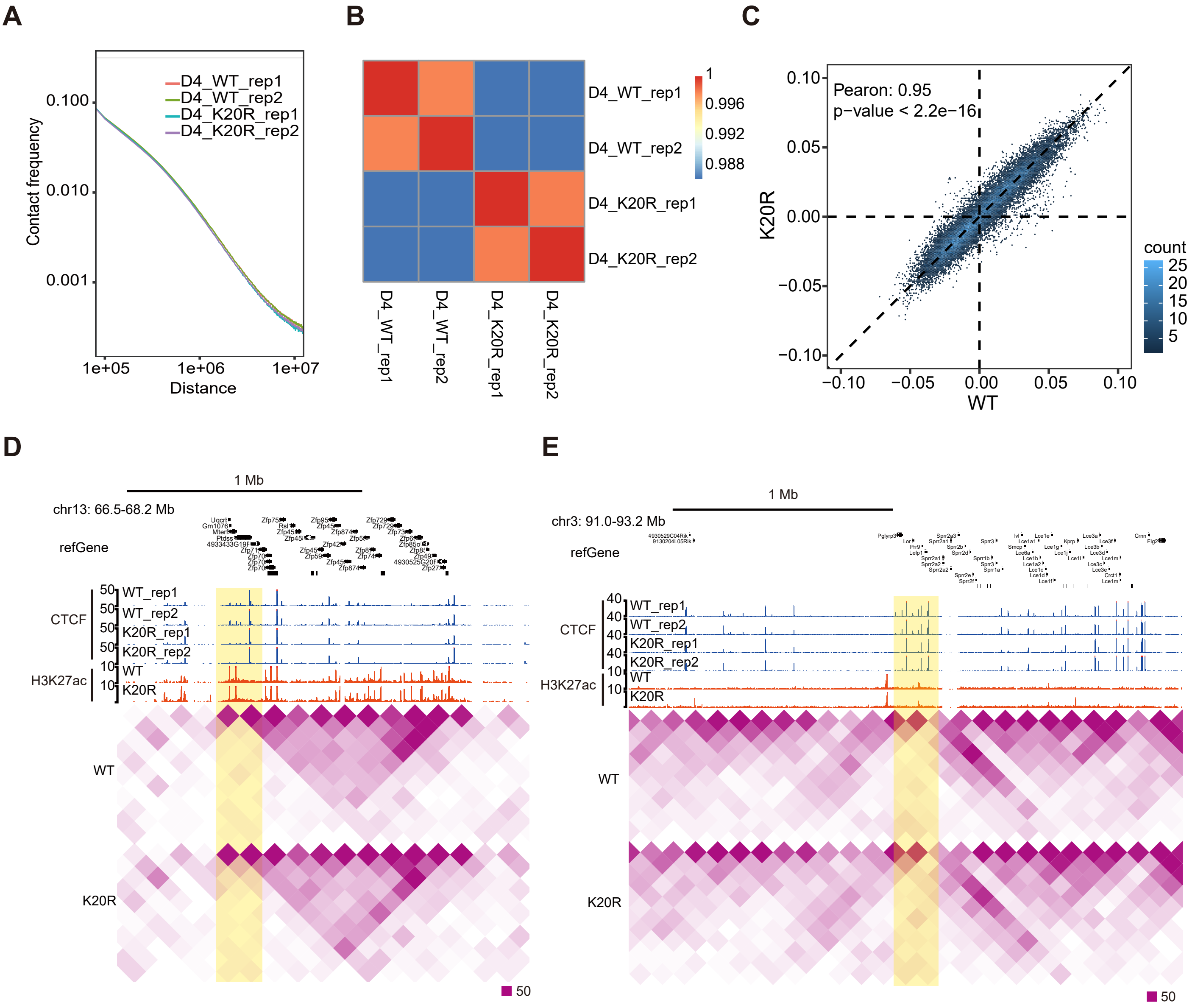

Supplementary Fig. 5

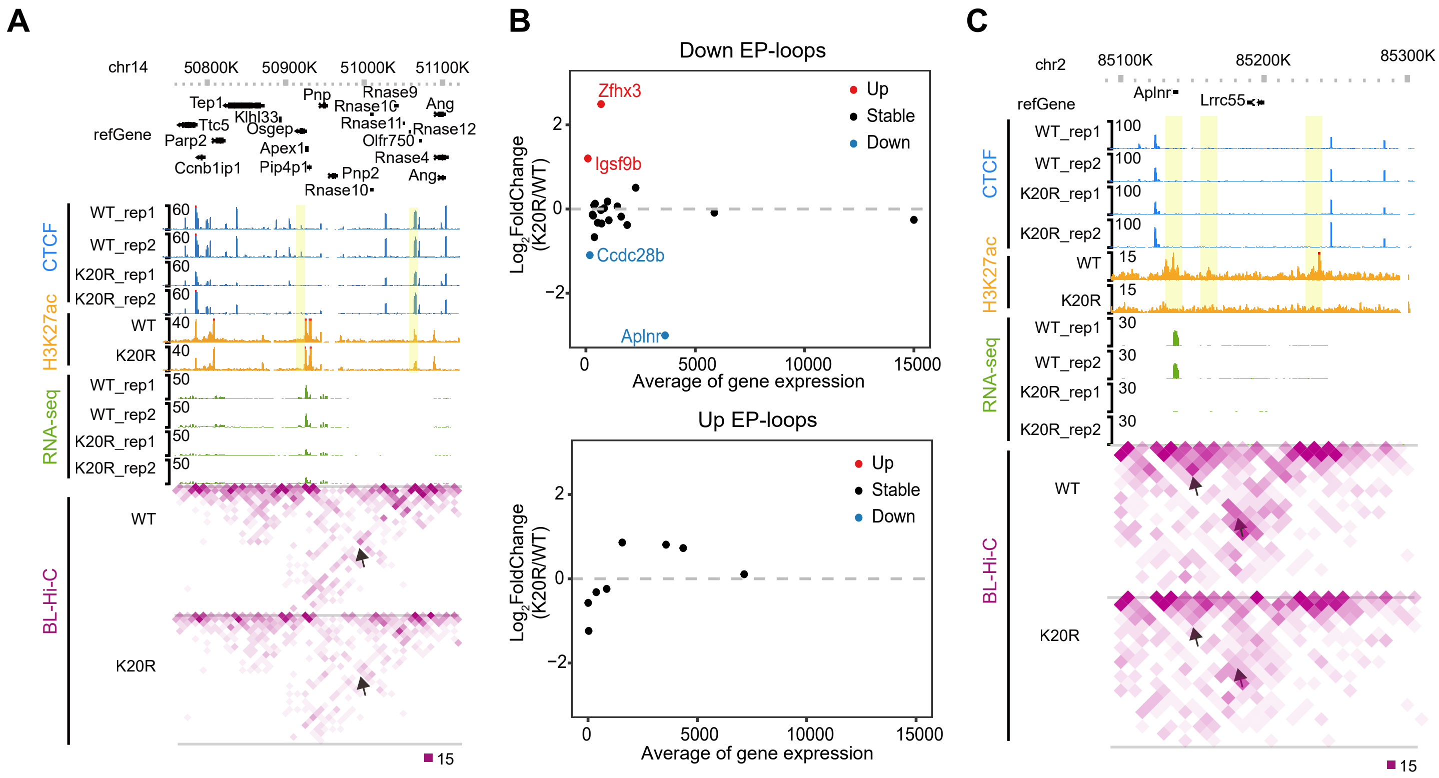

Supplement: Supplementary file 4 — Additional file 4. Supplementary figures [file 13619_2022_131_MOESM4_ESM.pdf]
